# Single-cell genomics for resolution of conserved bacterial genes and mobile genetic elements of the human intestinal microbiota using flow cytometry

**DOI:** 10.1080/19490976.2022.2029673

**Published:** 2022-02-07

**Authors:** Dylan Lawrence, Danielle E. Campbell, Lawrence A. Schriefer, Rachel Rodgers, Forrest C. Walker, Marissa Turkin, Lindsay Droit, Miles Parkes, Scott A. Handley, Megan T. Baldridge

**Affiliations:** a Department of Medicine, Division of Infectious Diseases, Washington University School of Medicine, St. Louis, MO, USA; b Edison Family Center for Genome Sciences & Systems Biology, Washington University School of Medicine, St. Louis, MO, USA; c Department of Pathology & Immunology, Washington University School of Medicine, St. Louis, MO, USA; d Division of Gastroenterology Addenbrooke’s Hospital and Department of Medicine, University of Cambridge, Cambridge, UK

**Keywords:** Single cell, single-amplified genome, genomic assembly, mobile genetic elements, prophage

## Abstract

As our understanding of the importance of the human microbiota in health and disease grows, so does our need to carefully resolve and delineate its genomic content. 16S rRNA gene-based analyses yield important insights into taxonomic composition, and metagenomics-based approaches reveal the functional potential of microbial communities. However, these methods generally fail to directly link genetic features, including bacterial genes and mobile genetic elements, to each other and to their source bacterial genomes. Further, they are inadequate to capture the microdiversity present within a genus, species, or strain of bacteria within these complex communities. Here, we present a method utilizing fluorescence-activated cell sorting for isolation of single bacterial cells, amplifying their genomes, screening them by 16S rRNA gene analysis, and selecting cells for genomic sequencing. We apply this method to both a cultured laboratory strain of *Escherichia coli* and human stool samples. Our analyses reveal the capacity of this method to provide nearly complete coverage of bacterial genomes when applied to isolates and partial genomes of bacterial species recovered from complex communities. Additionally, this method permits exploration and comparison of conserved and variable genomic features between individual cells. We generate assemblies of novel genomes within the *Ruminococcaceae* family and the *Holdemanella* genus by combining several 16S rRNA gene-matched single cells, and report novel prophages and conjugative transposons for both *Bifidobacterium* and *Ruminococcaceae*. Thus, we demonstrate an approach for flow cytometric separation and sequencing of single bacterial cells from the human microbiota, which yields a variety of critical insights into both the functional potential of individual microbes and the variation among those microbes. This method definitively links a variety of conserved and mobile genomic features, and can be extended to further resolve diverse elements present in the human microbiota.

## Introduction

The human intestine contains billions of microorganisms, collectively referred to as the microbiota^1^. While for over a century it has been recognized that these microbes play important roles in human health and disease,^2^ their specific activities, and indeed the identity of the players themselves, are still in the process of being uncovered. The evolution of techniques and technologies over the past two decades, especially the advent of next-generation sequencing, has enhanced our ability to understand and formally interrogate the contributions of the members of the microbiota and their functions in human health.^3^ However, even today up to 50% of the bacteria comprising the human gut microbiota lack a complete reference genome.^4^

Culturing of individual bacteria represents the classical approach to functionally characterize, and additionally obtain full genomes for, different taxa. While many members of the human intestinal microbiota have been cultured, many more have proven challenging to cultivate.^5^ One of the first culture-independent approaches developed for characterizing and studying bacterial communities was amplicon sequencing of the 16S rRNA gene.^6^ The 16S rRNA gene encodes for the structural RNA component of the small ribosomal subunit conserved in all bacteria, and its sequence has been used to assign taxonomy for several decades.^7^ The advent of high-throughput sequencing has facilitated the application of this technique to analyze complex communities including microbiota samples, providing a cost-effective means to describe bacterial communities in an unbiased way. However, 16S rRNA gene sequencing has numerous limitations, including failing to provide information on the remainder of the bacterial genome, and generally being insufficient to differentiate species- and strain-level differences in complex samples.^8^

The other frequently used approach for studying the microbiota, metagenomic sequencing, involves fragmenting and sequencing bulk DNA from microbial communities. Unlike 16S rRNA gene profiling or other amplicon-based approaches, metagenomics provides data from the entirety of the genomes present in a sample, and thus can be used for community-wide characterization of functional genes and pathways, estimated to include 3 million unique predicted genes in the gut.^9^ While metagenomics resolves functional differences not discernible by 16S rRNA gene analysis alone,^10^ it is ill-suited for fully delineating individual microbes, owing to fragmented assemblies, insufficient read depth, and masked genomic variation.^11–15^ Only recently tools have been developed to resolve strain-level differences within these metagenomic datasets.^16^

The genomes of gut microbes are highly variable, owing in part to the horizontal transfer of mobile genetic elements (MGEs),^17,18^ which include plasmids, bacteriophages, and conjugative transposons (CTns). Phages and CTns are key players in the transfer of functional genes, including antibiotic resistance, secretion systems and secreted toxins, and other pathogenic traits, between bacterial hosts in the intestinal microbiota.^19–23^ Further, the integration of MGEs into the host chromosome (*e.g*., as prophages) has profound implications for the behavior of intestinal microbes beyond the genes they carry.^24–30^ Functional understanding of the intestinal microbiota requires strain-level resolution of the variable and mobile components of bacterial genomes, which is impossible by 16S rRNA gene sequencing alone, and challenging with traditional metagenomics analyses.

An alternative means to rigorously define the individual members of complex microbial communities, including the human intestinal microbiota, is to apply physical separation methods. Microfluidic-based approaches have been applied to human microbiota samples to isolate single bacterial cells,^31^ and while originally developed for use on eukaryotic cells, a growing body of work has applied flow cytometry to isolate single microbial cells from environmental samples.^32–38^ Though flow cytometric approaches have been applied to human-associated samples previously,^39^ they have generally targeted populations of cells with specific properties, and have focused less on capturing single cells. More recently, single-cell isolation has been applied to human-derived bacteria to interrogate relationships between bacteria and bacteriophages in the human gut.^40^ While this method is an important advance, it focuses on previously known bacterial types, and does not further interrogate those cells representing unexplored diversity.

Here, we present an accessible method for isolating and analyzing genomes derived from single bacterial cells from human stool samples using fluorescence-activated cell sorting (FACS). We also present a sequencing and computational filtering pipeline that allows for informed selection of bacterial cells to focus sequencing efforts appropriately. Finally, we demonstrate that sequencing individual cells allows for discovery of genomic microdiversity and resolution of MGEs.

## Materials and methods

### *Development of an* Escherichia coli *reference genome*

Frozen *E. coli* stocks (GoldBio #CC-101-B) were scraped and transferred to 15 mL tubes filled with 5 mL Lysogeny broth (LB) and agitated overnight at 37°C. 1 mL of *E. coli* culture was transferred to a 2 mL Eppendorf tube containing homogenization beads. After homogenization DNA was extracted from these samples by phenol:chloroform extraction and cleaned with the DNeasy Blood and Tissue Kit (Qiagen 69504). DNA was prepared as outlined in the “Genomic sequencing” section below. Additional DNA was prepared for Oxford Nanopore sequencing. First, DNA was cleaned again with Qiagen Genomic-tip 100/G DNA Purification (Qiagen 10243), then it was run on a 0.8% TBE gel to determine approximate fragment size. Samples were barcoded with the Oxford Nanopore EXP-NBD104 native barcoding kit and prepared with the Oxford Nanopore SQK-LSK109 sequencing kit. Prepared DNA was then run on a MinION sequencer with MinION flowcell. Long-read sequences were processed with Guppy and hybrid assembly was performed with Unicycler.^41^

### Human stool samples

Human stool samples were obtained from a cohort of longitudinally sampled inflammatory bowel disease patients and their healthy household controls, collected from Addenbrooke’s Hospital, University of Cambridge, UK, and described previously.^42^ Patients 1, 4, and 6 were individuals with inflammatory bowel diseases: respectively, a female with Ulcerative Colitis, a male with Crohn’s disease, and another male with Crohn’s disease. Patients 2, 3, and 5 were household controls, and were, respectively, a healthy male, a healthy female paired with Patient 6, and a healthy female paired with Patient 1. Stool samples were removed from −80°C storage and immediately placed on dry ice. Samples were then cored using a small cork borer (Humboldt Manufacturing Company H9661). Core samples were transferred to 2 mL Eppendorf tubes containing homogenization beads. After homogenization, DNA for community profiling was extracted from these samples by phenol:chloroform extraction and cleaned with the DNeasy Blood and Tissue Kit (Qiagen 69504). DNA was prepared as outlined in the “16S rRNA gene sequencing and analysis” section below.

### *Sorting of* E. coli *and human stool samples*

Frozen *E. coli* stocks (GoldBio #CC-101-B) were scraped and transferred to 15 mL tubes filled with 5 mL Lysogeny broth (LB) and agitated overnight at 37°C. 1 mL of *E. coli* culture was transferred to a 1.5 mL Eppendorf tube. Samples were spun for 5 minutes at 10,000 g, supernatant was removed, and sample was resuspended in cold TE buffer. The cell pellet was then suspended in 1 mL Tris-EDTA (TE) buffer (Corning 46–009-CM).

Stool samples were stored at −80°C and transferred to dry ice where an ~100ug sample was cored into a 1.5 mL Eppendorf tube and suspended in 1 mL TE buffer. Samples were passed through a 35 μm cell strainer (Falcon 352235). Flow-through was collected and transferred back to a 1.5 mL Eppendorf tube. Samples were spun for 5 minutes at 10,000 g, supernatant was removed, and sample was resuspended in cold TE buffer. Samples were then split into 500uL aliquots. 1uL of SYTO-9 (Invitrogen S34854) was added to one of these samples and mixed by inversion. Samples were incubated in the dark on ice for thirty minutes. After incubation, samples were spun at 10,000 g for 1 minute, supernatant removed, and washed with 1 mL of cold TE buffer. This was repeated two additional times. Samples were then kept in the dark, on ice, while being transported to the flow cytometer. Samples were sorted on the BD Aria II-3 Flow Cytometer (Flow Cytometry and Fluorescence Activate Cell Sorting Core at Washington University School of Medicine) into 96-well plates containing 8uL of TE buffer. Negative controls were used to establish gating parameters for remove of small debris and doublets as well as to establish an unstained population. The top 10% brightest cells were selected and individually sorted into wells, then plates were immediately sealed and placed on dry ice before being transferred to a −80°C freezer.

### Flow cytometric analysis of bacterial cultures

Isolates of *E. coli* (GoldBio #CC-101-B), *Bifidobacterium adolescentis* (ATCC 15705), *Faecalibacterium prausnitzii* (two isolates kindly provided by Anne Rosen and Andy Kau), and *Subdoligranulum variabile* (DSMZ 15176) were cultured overnight. *B. adolescentis, F. prausnitzii*, and *S. variabile* were grown in an anaerobic chamber in Modified Reinforced Clostridial Broth at 37°C. All samples were centrifuged at 3000 g for 10 minutes, resuspended in 1 mL of PBS, and then normalized to 2.5 ODs. 1 mL of normalized culture was stained with 1uL of SYTO-9 nucleic acid and incubated in the dark for 30 minutes. After incubation, samples were centrifuged at 5000 g for 3 minutes, supernatant removed, and washed with 1 mL of PBS a total of 3 times. Samples were then analyzed on a Beckman-Coulter Cytoflex S.

### Amplification of bacterial cell DNA

Sorted bacterial cells were first lysed by adding 1uL of lysis solution (0.4 M KOH, 10 mM EDTA) to each well, then incubated for 30 minutes at 30°C. Lysis was stopped with the addition of 1uL of 10 mM Tris, pH 4.0. Subsequently, 5.2uL of DNase/RNase free water (Fisher Scientific AM9935), 2uL of NEB 10X Buffer (NEB B0269S), 1uL of Exo-resistant random hexamers (MCLAB ERRP-110), 0.5uL of Phi29 DNA polymerase (NEB M0269L), and 0.40uL of NEB BSA (B9000S, resuspended to 10 mg/mL) per well were combined and incubated at 30°C for 30 minutes. All reagents except the Phi29 DNA Polymerase and Exo-resistant primers were exposed to 10 minutes of UV treatment in a Stratalinker 2400 UV crosslinker prior to mixing. Immediately prior to addition to wells, 0.8uL of 10 mM dNTPs (Fisher Scientific PRU1515) and 0.1uL of 100X SYBR Green I (S7563) were added to this mixture, then the 10uL mix was added to each well. Plates were then sealed and briefly vortexed. Amplification was performed in a QuantStudio 3 Real-Time PCR machine (Applied Biosystems A28567). Samples were kept at 30°C for 7.5 hours with a reading of fluorescence intensity taken every 3 minutes. Enzymes were then heat-inactivated at 65°C for 10 minutes. DNA quantities after reaction were analyzed using the Qubit dsDNA HS Assay Kit (Invitrogen Q32854).

### 16S rRNA gene sequencing and analysis

For 16S rRNA gene sequencing, primer selection and PCRs were performed as described previously.^43^ Briefly, each sample was amplified in triplicate with Golay-barcoded primers specific for the V4 region (F515/R806), combined, and confirmed by gel electrophoresis. PCR reactions contained 18.8 μL RNase/DNase-free water, 2.5 μL 10X High Fidelity PCR Buffer (Invitrogen), 0.5 μL 10 mM dNTPs, 1 μL 50 mM MgSO4, 0.5 μL each of the forward and reverse primers (10 μM final concentration), 0.1 μL Platinum High Fidelity Taq (Invitrogen) and 1.0 μL genomic DNA. Reactions were held at 94°C for 2 min to denature the DNA, with amplification proceeding for 26 cycles at 94°C for 15s, 50°C for 30s, and 68°C for 30s; a final extension of 2 min at 68°C was added to ensure complete amplification. Amplicons were pooled and the presence of amplicon confirmed by gel electrophoresis. The amplicons were then purified with 0.6x Agencourt Ampure XP beads (Beckman-Coulter) according to the manufacturer’s instructions. The final pooled samples, along with aliquots of the three sequencing primers, were sent to the DNA Sequencing Innovation Lab (Washington University School of Medicine) for sequencing by the 2x250bp protocol with the Illumina MiSeq platform. 16S rRNA gene sequencing data have been uploaded to the NCBI BioProject database (BioProject ID PRJNA733504).

### Genomic sequencing of single cells

Amplified DNA was used for tagmentation using the Nextera DNA Library Preparation kit (Illumina) as previously described.^43^ This was followed by PCR-mediated adapter ligation using KAPA HiFi PCR master mix (Roche). The tagmented and indexed DNA libraries were selected for approximately 200bp size using AMPure XP magnetic beads. Equimolar quantities of libraries were run on the Illumina NextSeq platform using a paired end 2 × 150 protocol. Genomic sequencing data have been uploaded to the NCBI BioProject database (BioProject ID PRJNA733504).

### Analysis of SYBR green curves

Multicomponent data gathered during MDA amplification was exported from the QuantStudio Design and Analysis Software, then fed to a custom Python script^44^ making use of scikit-learn^45^ to predict values according to a sigmoidal curve. This data was then imported into R version 4.0.3.^46^ Samples were classified according to whether they produced a 16S rRNA gene amplicon band above our threshold of detection (10e3 copies) and had a kParameter greater than or equal to 0.1. Samples with kParameters above this threshold were considered to fit to the sigmoidal curve. Samples were classified into four categories based on whether they were 16S rRNA gene amplicon-positive or -negative and whether they fit to the sigmoidal curve. Samples within these categories were then compared by Fisher’s Exact Test. SYBR Green multicomponent plots were visualized with ggplot2^47^ in R.

## Analysis of 16S rRNA gene V4 amplicons

Read quality control and the resolution of amplicon sequence variants (ASVs) were performed with the dada2 R package.^48^ All reads had the first 10 bases and last 15 bases removed, creating a uniform set of 215 bp length reads. Taxonomy was assigned using the RDP (RDP trainset 18) 16S rRNA gene sequence database.^49^ Ecological analyses, such as alpha-diversity (richness, Shannon diversity), were performed using phyloseq and additional R packages.^50^

All reads derived from single-cells were then analyzed with a custom Python script^44^ which compared single-cell amplicons to those found in community profiles, removing any samples lacking a 100% match to an ASV in the community. Additionally, any samples that did not have a single ASV representing at least 70% of the total ASVs were also removed. Samples passing this filtering step were then imported with phyloseq for further analysis. Phyloseq objects were converted to a dataframe, converting all abundance numbers to percent abundance. Overall abundance profiles were then visualized with ggplot2.^47^ Single-cell ASVs derived from Patient 3 were further analyzed for recovery on a per-plate basis in R. ASV counts per-plate were generated in R and then compared by ANOVA and Tukey’s test.

### Mapping shotgun sequencing reads to reference genomes

Shotgun-sequencing reads were trimmed and quality filtered by bbtools^51^ and read sets were analyzed by FastQC.^52^ Reads were mapped to reference genomes identified by 16S rRNA or ReferenceSeeker^53^ using bowtie2^54^ with default settings. Mapping to references was visualized using a Python script^44^and seaborn.^55^ 16S rRNA gene locations were determined with barrnap.^56^ Origins of replication were identified with the Ori-Finder web server.^57^ Read-depths across the genome were derived with samtools.^58^ Statistical comparisons of site-depths were performed in R by ANOVA and Tukey’s test.

### Read and ASV rarefaction analyses

Read sets were subsampled to specified read counts using bbtools and mapped back to their reference with bowtie2 as with the full mapping. Read depths at each count were then analyzed in R with ANOVA and Tukey’s test. Unique ASV counts were analyzed in R with the phyloseq library. All combinations of plates were generated and examined for unique ASV counts. All plate count sets were then compared using ANOVA and Tukey’s test.

### Genome assemblies

Genome assemblies were performed with SPAdes^59^ set to single-cell mode. Contigs shorter than 1500 basepairs were removed from the set with bbtools. Assembly statistics were generated with QUAST^60^ and are summarized in Supp Table S1. Completeness and contamination metrics were generated using CheckM^61^ with the lineage-specific workflow. Assembly taxonomy assignments were performed with GTDB-Tk^62^ using the classify workflow. Co-assemblies of multiple single-cell genomes were visualized with Anvi’o.^63^ Genome assemblies have been submitted to the NCBI BioProject database (BioProject ID PRJNA733504).

### Phylogenetic analyses

16S rRNA genes were extracted from assemblies and reference genomes using barrnap. Target proteins were searched against assemblies and reference genomes using BLAST-P^64^ with an e-value cutoff of 0.05. Both protein and DNA sequences were aligned in MEGA-X^65^ using the MUSCLE^66^ algorithm. Phylogenetic trees were generated using Maximum-Likelihood estimation with 500 trees generated.

### Identification and analysis of prophage sequences

Contigs predicted by Cenote-Taker2^67^ to contain phage sequences were grouped according to their best BLAST-P hits for taxonomy. Within each predicted phage group according to BLAST-P hit, all pairwise average nucleotide identities were calculated with fastANI^68^ v1.3, and the contig with the longest predicted phage sequence was chosen as a representative for further analysis. Each representative phage-like contig was annotated by first calling ORFs with Prokka^69^ then combining results from BLAST-P against all amino acid sequences in the NCBI RefSeq Virus database, PhANNs,^70^ and Phyre2^71^ (Supp Table S3). Next, reads from non-representative predicted phage contigs were mapped to their representative contig with Bowtie2 using default parameters. Read mapping was normalized to the total number of reads after trimming for each sample, and converted to a coverage curve. Finally, each representative phage-like contig was used as a query to search for related regions of all complete genome assemblies available on NCBI GenBank or RefSeq for the genus *Bifidobacterium* (n = 224 genomes) or *Faecalibacterium* (n = 9 genomes) by BLAST-N with default settings. Blast hits with percent identities greater than 98% and longer than 14 kb were considered significant hits. Genomes with significant hits were trimmed to include the region containing the significant BLAST-N hit and 5 kb of flanking sequence. These trimmed regions and their respective phage-like contigs were aligned by Progressive Mauve with default settings.^72^

### Network analysis of conjugative transposons

Contigs predicted by Cenote-Taker2 to contain CTns were used to generate the network of CTns. First, a reciprocal nucleotide BLAST was performed to compare all CTn predictions to each other, and the total percent length aligned (PLA) for every pair is calculated by summing the length of non-overlapping hits. PLAs greater than or equal to 20% were passed to MCL^73^ v14.137 with an inflation parameter of 2, generating CTn clusters. The network was visualized with Cytoscape^74^ v3.8.2.

## Results

### *Single-cell analysis of* E. coli *yields near-complete genomes*

**Figure 1.**
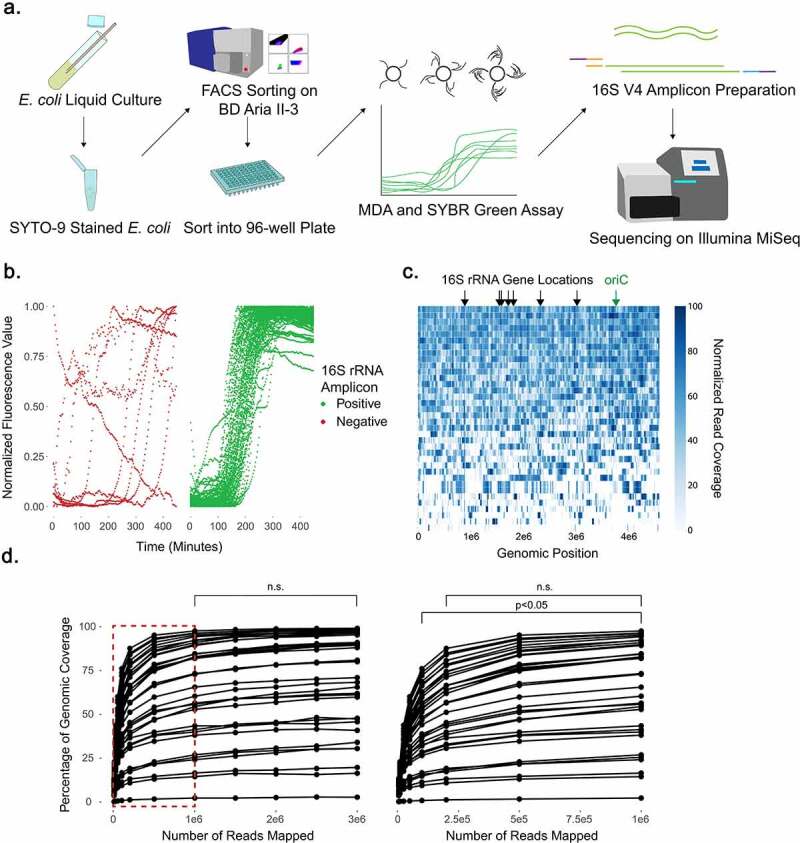
Near-complete E. coli genomes can be obtained from single cells, though increasing sequencing depth fails to resolve gaps in coverage. (a) Schematic for single-cell sorting and analysis of E. coli. Cells were stained with SYTO-9 nucleic acid stain, sorted, and then amplified by multiple displacement amplification (MDA) in a reaction tracked with SYBR Green nucleic acid stain. The V4 region of the 16S rRNA gene was amplified, and 16S V4 amplicon-positive samples were further shotgun sequenced on the Illumina MiSeq platform. (b) SYBR Green fluorescence values during MDA, grouped by subsequent positive or negative 16S V4 PCR result. (c) Read coverage of 36 individual cells mapped against the reference genome by bowtie2. Read coverage displayed as a heatmap with each bp represented. Overall genomic coverage ranged from 4.1% to 99.6%. Locations of seven 16S rRNA genes and origin of replication (oriC) on the genome are indicated. (d) Comparison of genomic coverage to number of reads used for mapping. Reads were subsampled from the original read set using bbtools and re-mapped by bowtie2 to the reference genome. Subsampling and re-mapping were performed 10 times for each sample to a maximum of 3e6 reads per cell. Statistical comparison at all depths was performed by ANOVA and Tukey’s test; n.s. = non-significant. The boxed region in the left plot is shown expanded on the right.

We adapted our method for single-cell isolation based upon flow cytometric approaches developed for environmental samples.^33,75^ We initially applied our sorting approach to *Escherichia coli* DH5α, a common laboratory strain, to determine how well we could resolve the genomic sequence of single cells for a strain with a matched reference genome. We first used DNA extracted from culture to sequence and generate in-house a full-length, single-contig reference genome for *E. coli* DH5α, utilizing both short-read Illumina sequencing and long-read Nanopore sequencing for hybrid assembly. *E. coli* cultures were then stained with SYTO-9 nucleic acid stain to identify intact bacterial cells by fluorescence,^76^ and individual bacterial cells were deposited into 96-well plates containing lysis buffer (Figure 1(a)). To amplify the 2 to 8 femtograms of bacterial DNA present in each well from an individual cell, we performed multiple displacement amplification (MDA), which utilizes the bacteriophage-derived Phi29 polymerase to exponentially and non-specifically amplify DNA with high fidelity.^77^ MDA was performed in the presence of SYBR Green to permit monitoring of DNA amplification by fluorescence, then PCR for the V4 region of the 16S rRNA gene was performed on all wells, serving as an initial filter to identify wells into which a bacterial cell had been deposited and amplified. We found a correlation between SYBR Green sigmoidal fluorescence curves with threshold values between 100 and 300 minutes and subsequent positive 16S V4 PCR, here defined as appearance of a band by gel electrophoresis analysis (P < 0.001 by Fisher’s exact test) (Figure 1(b)), showing predictive value in evaluation of these curves prior to subsequent gel electrophoresis analysis. The limit of detection of our 16S V4 PCR was determined to be 10e3 copies (Supp Figure S1).

Thirty-six individual *E. coli* cells were then selected at random from among the 16S V4 PCR-positive wells for shotgun sequencing. Libraries were prepared from the amplified DNA using a Nextera transposase method^43^ and paired-end sequencing performed on an Illumina MiSeq platform. We obtained a range of ~3.5–18.7 million 150-bp reads per cell. Based on our *E. coli* reference genome length of 4,586,346 bp, this gives a theoretical maximum depth of coverage between 113 and 610x, well above a 50x coverage minimum,^78^ though we did not expect to see these coverages reflected across the entire target genome due to the exponential amplification caused by MDA.^79^

We mapped reads onto our reference genome and found the genome coverage to be variable, ranging from 4.1% to 99.6% coverage (median coverage of 84.51%) with an average read depth of 207x (Figure 1(c,d)). We then assessed *de novo* assemblies of all cells using CheckM,^61^ which revealed completeness scores between 0.0% and 99.2% with a median of 74.1% (Supp Table S1). A reference *E. coli* genome (GCF_000005845.2) and our in-house assembled *E. coli* genome both yielded completeness scores of 99.97%, as did a co-assembly of reads from all 36 *E. coli* cells. Contamination scores for individual cells ranged from 0.0% to 1.7%, with an average of 0.2%, in comparison to contamination scores of 0.04% for GCF_000005845.2 and our in-house assembled *E. coli* reference genomes (Supp Table S1). We assessed whether SYBR Green threshold values were correlated with genomic coverage, and found a significant association between lower threshold and improved coverage, further supporting their predictive value (Supp Figure S2). Comparing coverage across all cells did not reveal regions that were preferentially amplified. We explicitly compared normalized read-depths at the origin of replication and the 16S rRNA genes to the remainder of the bacterial genome as two hypothetical regions of differential coverage, but found no significant enrichment at these sites (P = 1.00 and P = 0.79 respectively by Tukey’s test) (Supp Figure S3). To determine if read depth was a limiting factor in our overall genomic coverage, we performed rarefaction analysis, subsampling reads beginning at 5,000 reads and then increasing to 10,000, 25,000, 50,000, 100,000, 200,000, 500,000 and then an additional 500,000 up to 3,000,000 reads, and then re-mapping. While increasing read counts improved the overall genomic coverage (Figure 1(d)), increased sequencing depth exhibited diminishing returns: between 100,000 to 1,000,000 reads genomic coverage increased 20.5% ± 8.5% (0.023% per 1000 reads), but from 1,000,000 to 3,000,000 coverage increased only 5.3% ± 2.3% (0.0026% per 1000 reads), a non-significant improvement to coverage (P = 1.00 by Tukey’s test). Importantly, genomic coverage of cells with poor coverage (e.g. 4.1%) was not substantially improved with greater read depth. We thus elected to subsequently aim for a minimum read depth of 1,000,000 reads per cell as a means to increase the number of cells that could be analyzed per sequencing run by multiplexing additional cells while still obtaining optimal recovery of genomic features.

### Single-cell sorting of human microbiota samples recovers diverse taxa

**Figure 2.**
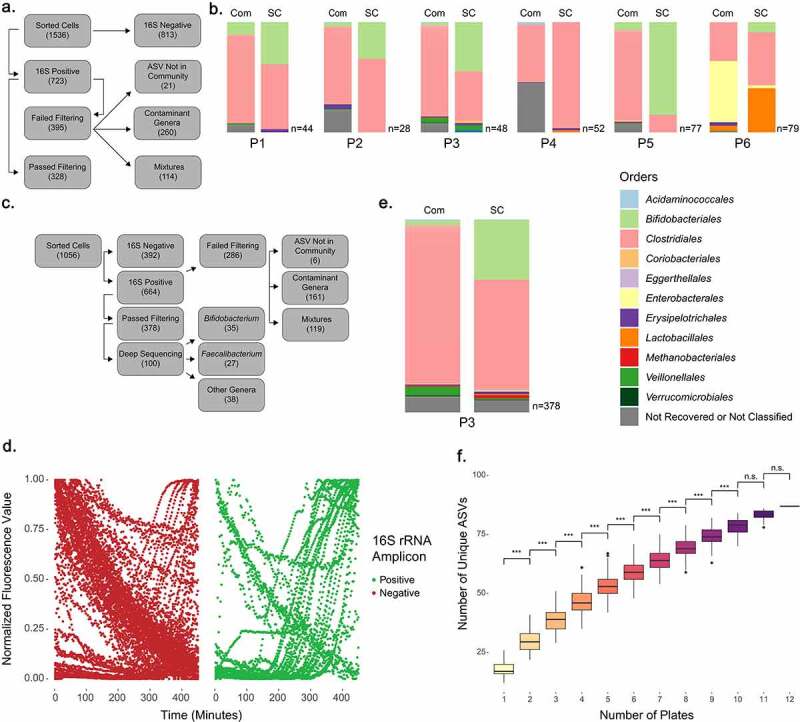
16S analysis of sorted single cells reveals recovery of abundant and rare bacterial taxa from human microbiota samples. (a) Flowchart showing fate of 1536 cells sorted from P1 through P6. (b) Comparison of V4 amplicon sequencing results for community taxonomic profiles (Com) and single cell taxonomic profiles (SC) for six patient samples, with number of single cells per sample denoted (n = 28–79 per sample). (c) Flowchart showing fate of 1056 cells sorted from P3. (d) SYBR Green fluorescence values during MDA, grouped by subsequent positive or negative 16S V4 PCR. (e) V4 amplicon sequencing results from community taxonomic profile (Com) and taxonomic profile of additional single cells (SC) from P3 sample. (f) Comparison of unique ASVs found as compared to the number of 96-well plates sorted. Analyzed by Tukey’s test (*** denotes P ≤ 0.001).

We next applied our single-cell sorting approach to six human stool samples obtained from a cohort of patients with inflammatory bowel diseases (n = 3) and their healthy household controls (n = 3).^42^ We sequenced the V4 region of the 16S rRNA gene on bulk DNA extracted from each of these samples to define the community composition, and then sorted 1536 individual bacterial cells across the six samples (Figure 2(a)). Analysis of 16S rRNA gene V4 amplicons from single cells after MDA revealed 723 16S-positive cells (47.1% of sorted cells), which underwent genomic sequencing. To rigorously exclude any environmental contaminants or wells with multiple cells present,^80^ we developed a stringent filtering pipeline. First, we trimmed and processed all single-cell V4 amplicons using dada2^48^ and discarded any samples that did not contain a single amplicon sequence variant (ASV) representing more than 70% of the total recovered ASVs in the well. We then compared this ASV to all ASVs in the community profile of the sample from which the cell was sorted, and kept only samples with 100% identity to an ASV in the community profile.^81^ 328 single cells passed all filtering criteria (45.4% of 16S V4 amplicon-positive samples), a recovery rate similar to other single-cell approaches that have been applied to bacterial samples^32,40^ (Supp Table S2). We assessed the 395 16S rRNA gene V4 amplicon-positive samples which failed our filtering pipeline to determine their potential source(s). 260 (65.8% of positive cells) of these samples belong to bacterial genera which are known kit contaminants^82–85^ (Supp Table S2) and contained an ASV absent from the community profile. 114 (28.9% of positive cells) of these samples lacked a single ASV representing ≥70% of total representation in the sample. A subset of these “mixtures” may be true positives, containing ASVs from multiple 16S sites with single nucleotide polymorphisms, which were not sampled in our community profile reference. Finally, the remaining 21 (5.3% of positive cells) cells represented bacterial families normally present in the intestinal microbiota, but containing ASVs not found in the community. These may represent rare taxa that were not detected by community profiling. We elected to remove these samples to ensure only verified single cells would be analyzed.

Cells passing filtering were then compiled on a per-sample basis, and compared to the original community profiles (Figure 2(b)). Analysis of the recovery of different taxa revealed statistically significant skewing of the singly sorted cells toward *Bifidobacteriales* (4.07% ± 4.07 in the original communities versus 32.31% ± 26.86 of sorted cells; P < 0.05 by ANOVA). We speculated that our selection of the 10% most brightly SYTO-9-stained cells may account for this bias, and indeed it has been recently reported that nucleic acid stains can mediate differential fluorescence intensities in different bacterial species.^86^ We assessed the fluorescence of isolates of *Bifidobacterium adolescentis, Faecalibacterium prausnitzii, and Subdoligranulum variabile* compared to *E. coli* in the presence and absence of SYTO-9 staining. We found that both *B. adolescentis and F. prausnitzii* exhibited greater mean fluorescence intensity than *E. coli* when stained with SYTO-9 (Supp Figure S4). We also examined the original location of filtered single cell samples on each plate (Supp Figure S5). While cells are deposited in row order, we did not observe obvious grouping of similar taxa along rows. Our approach captured sample-derived single bacterial cells representing 94.4% of the bacterial orders present in the community microbiota profiles for these samples, supporting the capacity of this method to obtain single cells of diverse taxa.

### In-depth analysis of single-cell genomes derived from a single patient

Following our initial survey of six patient samples, we selected the sample from Patient 3 (P3) for further analysis as its community profile had the highest alpha diversity of the six samples by all metrics measured (Supp Figure S6). We sorted 1056 cells from this sample following the same method employed in the initial survey. 664 wells were 16S-positive (62.9%), and of those, 378 passed our filtering pipeline (35.8% of total samples, 56.9% of 16S positive samples) (Figure 2(c)). We examined a subset of the SYBR Green curves generated during MDA, and similar to our observations with *E. coli* found there was a significant association between the presence of sigmoidal curves and subsequently 16S-positive samples (P < 0.001 by Fisher’s exact test) (Figure 2(d) and Supp Figure S7). We also determined that 16S-positive samples demonstrated an earlier inflection point in their sigmoidal curves, indicating earlier amplification (Supp Figure S8).

We compared the 378 recovered single cells to the original abundance profile of the community sample, and again observed a bias toward *Bifidobacteriales* (30.95% of single cells versus 2.86% of the community profile) (Figure 2(e)). The original community contained 24 unique orders, with 18 rare orders together representing just 6.8% of the community (Supp Table S2). With our single-cell approach, we recovered the six most common, as well as 5 of those orders present at <1%, for a total of 11 unique orders of bacteria from this sample. To determine if sorting of additional cells would improve recovery, we examined the number of unique ASVs we recovered from each plate and compared these between all possible combinations of plates sorted (Figure 2(f)). This revealed a significant difference in the number of unique ASVs we recovered with each plate sorted, suggesting that increased sampling may continue to reveal additional rare bacterial taxa in the absence of enrichment.

We selected approximately a third (100 total) of our recovered cells to undergo shotgun sequencing, selected from three subsets: (1) ASVs classified to the genus *Bifidobacterium*, (2) ASVs classified to the genus *Faecalibacterium*, and (3) those classified to genera which were either rare within the original sample (<2% of community profile) or belonging to genera with no current reference genomes in the RefSeq database. *Bifidobacterium* and *Faecalibacterium* were chosen for analysis as they represented the two most abundant recovered genera. Samples were sequenced with an average of 6,502,779 ± 4,563,731 reads. Bacterial genomes range in size from 500 kilobases to larger than nine megabases.^87^ To conservatively estimate our average genomic coverage, we chose to use seven megabases as a “standard” size for our bacterial genomes. With this estimation, our average maximum theoretical coverage for each cell was ~139x.

### *Analysis of* Bifidobacterium *single cells reveals variable genomic coverage and microdiversity*

We chose to leverage the abundance of *Bifidobacteria* represented in our single cells to explore the genomic diversity of this important and prominent gut-associated genus. Of the 35 cells classified in the genus *Bifidobacterium* by 16S V4 amplicon analysis, we identified four unique ASVs represented by these single cells, confirmed using both the Ribosomal Database Project (RDP) 16S V4 and the RefSeq 16S rRNA gene amplicon databases.^49,88^ The majority of single cells shared an ASV corresponding to *B. adolescentis* (n = 25), while other ASVs identified included *B. pseudocatenulatum* (n = 6), *B. longum* (n = 3), and a separate unique *B. adolescentis* ASV (n = 1).

**Figure 3.**
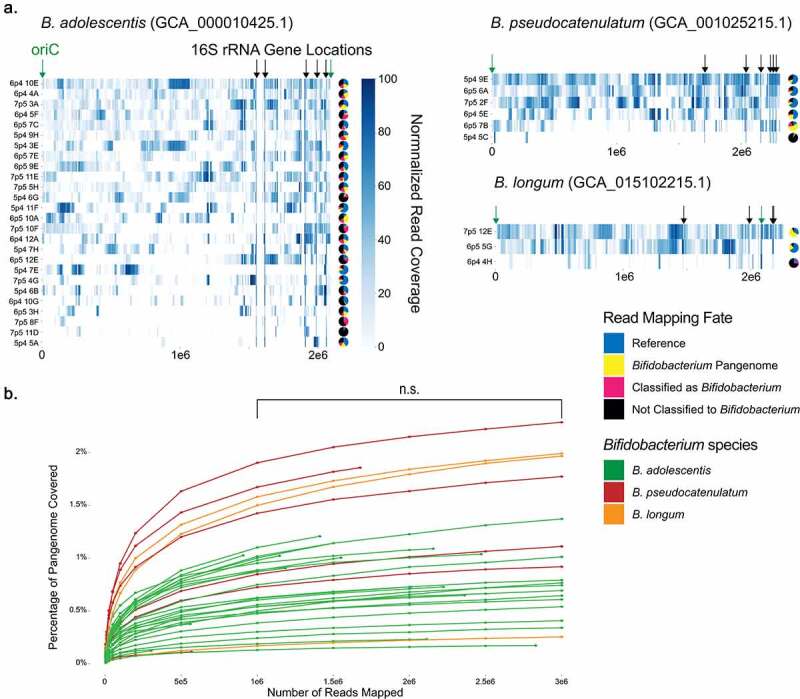
Analysis of *Bifidobacterium* single cells reveals variable genomic coverage. (a) Read coverage of 35 individual cell reads mapped against 16S rRNA gene V4 amplicon-matched reference genomes by bowtie2. Read coverage displayed as a heatmap with each bp represented. Overall genomic coverage ranged from 1.3% to 82%. Locations of 16S rRNA genes and origin of replication (oriC) on the genomes are indicated. Pie charts represent the proportions of reads for each sample as mapped to the reference genome, mapped to the *Bifidobacterium* pangenome, classified by MMSeqs2 as *Bifidobacterium*, or classified by MMSeqs2 as another taxon or left unclassified. (b) Rarefaction curve of *Bifidobacterium* pangenome coverage for each single-cell sample at increasing read depth. Between 1,000,000 and 3,000,00 reads there was no significant improvement in genomic coverage (p = 0.91 by Tukey’s test.).

Single-cell genomic reads were mapped to the most closely related reference genome, selected based upon the 16S rRNA gene V4 amplicon assignment (Figure 3(a)). As with *E. coli* single cells, read mapping was highly variable between samples with the total percentage of reads mapped ranging from 0.042% up to 70.1% (average of 33.9%). The percentage of genome coverage was also highly variable, ranging from 1.3% to 82% (average of 34.3%), and was positively correlated with the percentage of reads mapped (Supp Figure S9). Genomic coverage after read-based mapping of *Bifidobacterium* single cells to their associated reference genomes was lower compared to *E. coli* (34.3% versus 72.7%), an expected outcome based on the use of non-sample-derived reference genomes. Analysis of normalized read-depths revealed significantly higher coverage of the 16S rRNA genes in *B. adolescentis* in comparison to the overall bacterial genome (P = 0.02 by Tukey’s test), something not observed in *E. coli* (Supp Figure S10). We suggest that this is due to the sequence conservation of the 16S rRNA genes between sorted samples cells and their reference genomes, compared to the remainder of the genome, which is variable due to natural genome diversity within bacterial species. Finally, we examined whether identification of a reference genome using an alternate method would yield improved genomic coverage, and instead selected bacterial genome references with ReferenceSeeker.^53^ However, we found similar genomic coverage when mapping single-cell reads to references identified by ReferenceSeeker as to 16S rRNA-based references (Supp Figure S11).

To further define the source of these unmapped reads, we first mapped all reads for each single cell to a pangenome of 55 *Bifidobacterium* reference genomes.^89^ Alignment to other *Bifidobacterium* genomes accounted for an additional 0.4% to 59.3% (average of 14.9%) of the reads for each cell (Figure 3(a)). Rarefaction analysis performed on these samples supported that gaps in genomic coverage were not due to a lack of sequencing depth (Figure 3(b)). For the remaining reads that did not map to the *Bifidobacterium* pangenome, we used MMSeqs2, which assigns taxonomy by searching against protein reference databases, to compare these against the Genome Taxonomy Database (GTDB).^90–92^ MMSeqs2-based analysis indicated that an additional 0.1% to 35.5% (average of 12.8%) were *Bifidobacterium*-derived reads (Figure 3(a)). A remaining 6.1% to 94.1% (average of 38.4%) of reads were either classified to another microbe or remained unclassified (**Supplemental Data Sankey Diagrams**). These analyses suggested the possibility of greater diversity within our sample among single cells classified as *Bifidobacterium* than revealed by V4 amplicon analysis, but also raised the important caveat of putative contamination.

**Figure 4.**
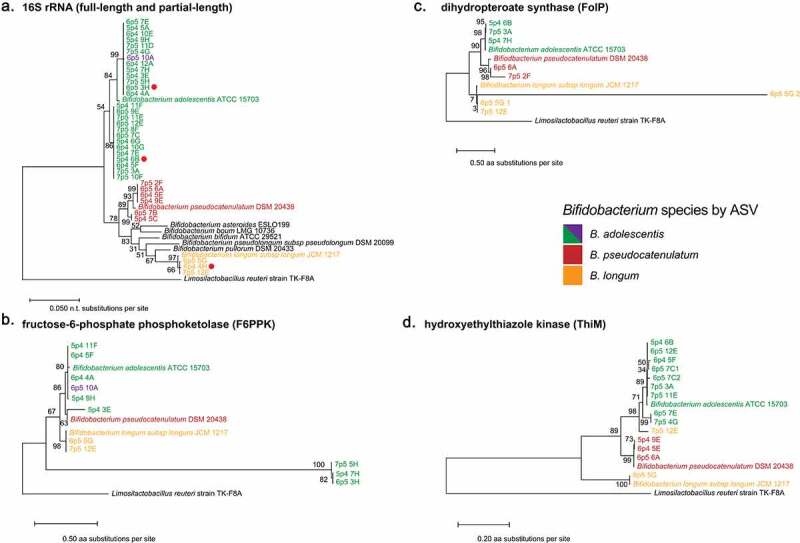
Analysis of *Bifidobacterium* full length 16S rRNA genes and conserved proteins reveals discordant microdiversity. (a) Phylogenetic tree of full-length 16S rRNA gene sequences, extracted by barrnap. Red dots represent sequences that were not full-length. (b-d) Gene trees of (b) fructose-6-phosphate phosphoketolase (F66PK), (c) dihydropteroate synthase (FolC), and (d) hydroxyethylthiazole kinase (ThiM) protein sequences derived from assembled single cells and indicated reference genomes. All proteins were derived from Prodigal predicted proteins searched with BLAST at an e-value of 0.05. All sequences were aligned in MEGA-X using MUSCLE and Maximum-Likelihood trees were generated in MEGA-X.

We then performed *de novo* assembly of all reads from each sample using SPAdes set to single-cell mode to generate a contig set for each single cell.^59^ All contig sets were then filtered to remove contigs smaller than 1500 basepairs in length, a moderately stringent threshold previously used for other assembly applications.^93–95^ CheckM analysis revealed genome completeness between 0.0 and 76.77% (average of 22.89%) with an estimated contamination of 0.0 to 0.46% (average of 0.06%) (Supp Table S1). The reference *Bifidobacterium* genomes all had 100% completeness but interestingly the references for *B. pseudocatenulatum* and *B. longum* had contamination scores of 1.14% and 1.23%, respectively. Each genome was then assigned taxonomy by GTDB-Tk,^62^ which was 77% concordant with 16S amplicon assignments, as all genomes were either classified as *Bifidobacterium* or were unable to be classified. To further compare these single cells against their associated reference genomes, we extracted the full-length 16S rRNA genes from all single cell assemblies, as well as from reference genomes used for mapping and reference strains used in prior *Bifidobacterium* phylogenetic analyses.^89^ While V4 amplicon assignments produce consistent clades with full-length 16S rRNA gene results in the assemblies, full-length 16S rRNA gene trees identify additional diversity within these clades that is not represented by the V4 amplicon sequencing (Figure 4(a)). We examined the V4 regions in the extracted full-length 16S rRNA genes and confirmed 100% identity between these regions and the previous V4 amplicon results.

To begin to assess the degree of microdiversity present between our individual cells, we selected a conserved *Bifidobacterium* gene encoding fructose-6-phosphate phosphoketolase (F6PPK), an enzyme essential for carbohydrate fermentation in *Bifidobacterium*,^96^ for further analysis. While we were able to extract only 5 full-length and 6 partial F6PPK genes, the estimated phylogenetic tree for F6PPK exhibits differential topology compared to the full-length 16S rRNA gene trees (Figure 4(a,b)). For example, while 16S rRNA gene analysis indicates that single cells 7p5 5H, 5p4 7H, and 6p5 3H are closely related to 6p4 4A, they encode divergent F6PPK genes compared to all other sequences, including references (Figure 4(a,b)). We then selected two additional proteins of interest in the *Bifidobacterium* genus, dihydropteroate synthase (FolP)^97^ and hydroxyethylthiazole kinase (ThiM),^98^ which are essential steps in *de novo* synthesis of vitamins folate and thiamine, respectively. As with F6PPK, we found that phylogenetic trees for ThiM and FolP are concordant with 16S rRNA gene V4 assignment, but discordant in comparison to the phylogenetic relationships indicated by the full-length 16S rRNA genes (Figure 4(a,c,d)). We find that FolP from 6p5 6A and 7p5 2F are distantly related, while in the 16S rRNA gene tree they share a clade (Figure 4(a,c)). ThiM is perhaps even more intriguing, indicating that the ThiM from 7p5 12E is highly divergent from other *B. longum* copies, instead sharing a recent common ancestor with ThiM from *B. adolescentis* (Figure 4(d)). These data support the use of single cell sequencing to obtain insights into the strain-level genomic variation present among individual microbes in complex communities.

### *Analysis of sorted* Faecalibacterium*-like cells reveals microdiversity and novel Ruminococcaceae genomes*

**Figure 5.**
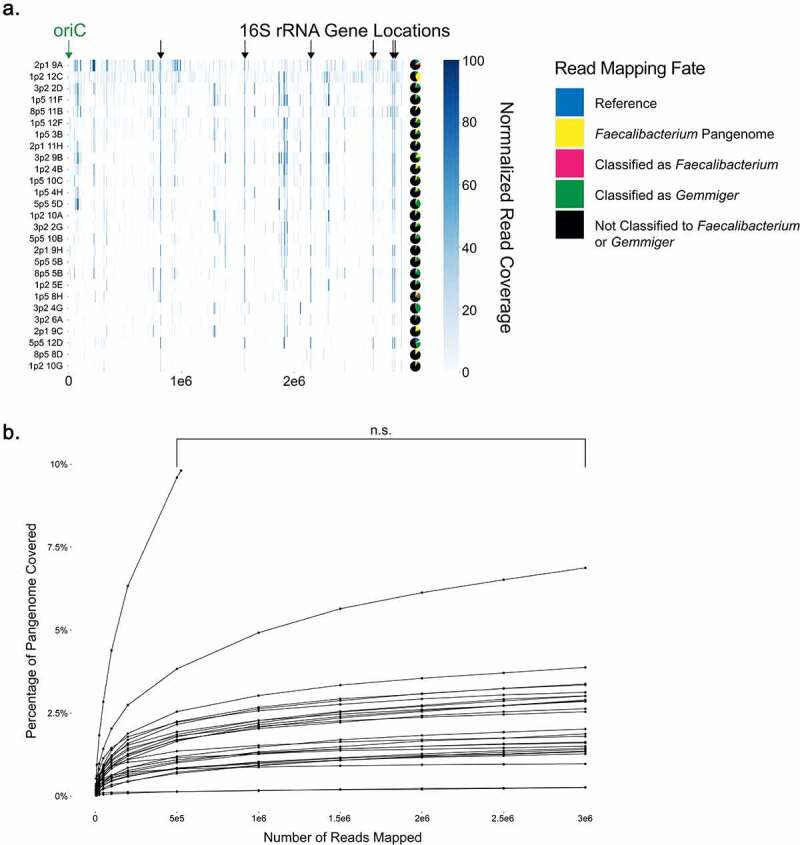
Analysis of *Faecalibacterium* single cells reveals limited coverage of available reference genomes. (a) Read coverage of individual cell reads mapped against *F. prausnitzii* reference genome by bowtie2. Read coverage displayed as a heatmap with each bp represented. Overall genomic coverage ranged from 1.1% to 16%. Locations of 16S rRNA genes and origin of replication (oriC) on the genomes are indicated. Pie charts represent the proportions of reads for each sample as mapped to the reference genome, mapped to the *Faecalibacterium* pangenome, classified by MMSeqs2 as *Faecalibacterium* or *Gemmiger*, or classified by MMSeqs2 as another taxon or left unclassified. (b) Rarefaction curve of read counts versus increasing read counts against the *Faecalibacterium* pangenome. Between 500,000 and 3,000,000 reads there was no significant improvement in genomic coverage (P = 0.78 by Tukey’s test.).

Similar to our approach with *Bifidobacterium*, we shotgun-sequenced 27 cells for which the 16S rRNA gene V4 amplicon assignment was to the genus *Faecalibacterium*. The genus *Faecalibacterium* contains only one assigned species, *F. prausnitzii*, which we used as our reference genome.^99^ Between 0.016% and 15.2% (average of 3.4%) of reads were mapped to the reference (Figure 5(a)), with lower genomic coverage than observed with our *Bifidobacterium* mapping, ranging from 1.1% to 16% (average of 4.7%). We compared the genomic coverage with percentage of reads mapped and found a correlation between these (Supp Figure S12). We also observed that the depth of coverage of the 16S rRNA genes in the reference genome was significantly increased compared to the overall genome (P = 0.00 by Tukey’s test) (Supp Figure S13). In sum, these data suggest that though our samples share a similar 16S rRNA gene with the reference, *Faecalibacterium* cells isolated from our sample are distantly related to the available reference genome.

We generated a pangenome containing six complete *Faecalibacterium* genomes and used this to map those reads that did not map to the reference. We found that only 1.3% to 33.5% (average of 7.3%) of total reads were further assigned in this fashion (Figure 5(a)). Rarefaction analysis against the pangenome indicated no significant difference in coverage between 500,000 and 3,000,000 reads per cell (P = 0.78 by Tukey’s test), suggesting that depth of coverage did not account for this limited read assignment (Figure 5(b)). Remaining reads were then compared to GTDB by MMSeqs2. An additional 0.002% to 7.0% (average of 1.3%) of reads were classified as *Faecalibacterium*. Interestingly, an additional 0.003% to 37.9% (average of 10.8%) of reads that failed to map to *Faecalibacterium* were classified to the genus *Gemmiger*, a recently reclassified taxon also in the family *Ruminococcaceae* with no full-length and few high-quality genome assemblies deposited to public databases. Finally, 54.9% to 93.5% (average of 77.2%) of reads classified to other microbes or were left unclassified.

We next assembled contigs for each single cell, and performed genome completeness assessments with CheckM, which yielded completeness between 0.0 and 72.14% (average of 24.32%) and contamination of 0.0 to 31.66% (average of 1.52%) (Supp Table S1). Reference genomes for *Faecalibacterium* and *Gemmiger* had completeness of 100% and 99.32%, respectively. Taxonomy was assigned with GTDB-Tk that had a concordance of 78% with 16S amplicon assignments. Similar to *Bifidobacterium*, nearly all cells not classified to the genus *Faecalibacterium* or *Gemmiger* were unable to be classified. One cell, 2p1 9H, was classified to the genus *Mediterraneibacter* and had only 5.34% contamination, suggesting that the 16S sequence corresponding to *Ruminococcaceae* may have been a contaminant in this well. Analysis with MMSeqs2 against the GTDB taxonomic database also revealed this discrepancy.

**Figure 6.**
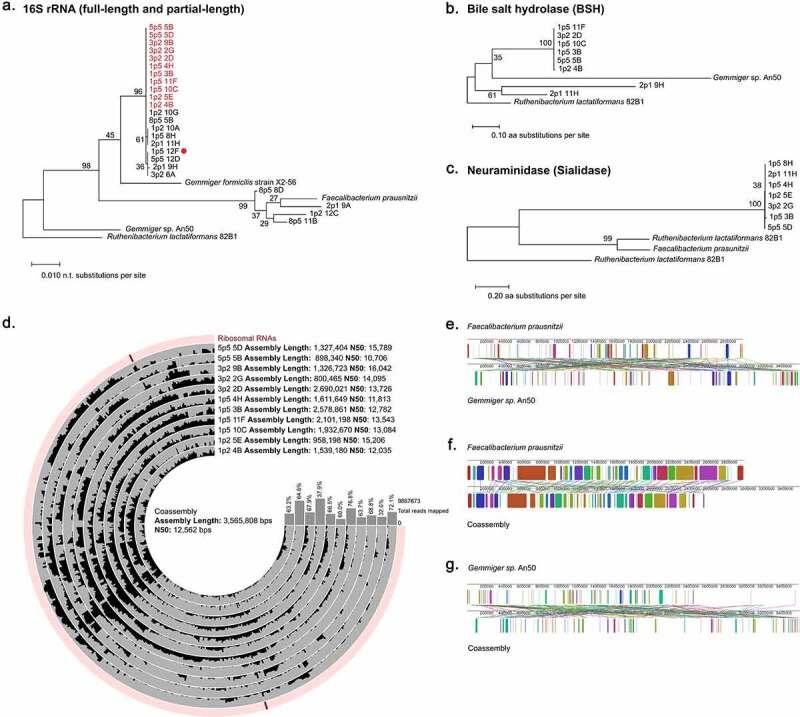
*Faecalibacterium* single cell assemblies reveal microdiversity and a novel *Ruminococcaceae* genome. (a) Phylogenetic tree of full-length 16S rRNA gene sequences extracted by barrnap with the exception of *Gemmiger formicilis* X2-56 which was obtained from RefSeq [NR_104846.1]. Red dots represent sequences that were not full-length. Sample names highlighted in red were used in the coassembly. (b,c) Gene trees of (b) bile salt hydrolase (BSH) protein sequences and (c) neuraminidase/sialidase protein sequences derived from single cell assemblies and reference genomes. All proteins were derived from Prodigal, predicted proteins searched with BLAST. All sequences were aligned in MEGA-X using MUSCLE and Maximum-Likelihood trees were generated in MEGA-X. (d) Coverage plot showing mapping of reads from 11 single cells classified as *Gemmiger* by MMSeqs2 (shown in red in A) mapped onto a coassembly of all reads. Assembly statistics and percentage of total reads mapped to the coassembly are displayed. (e-g) Alignments of (e) *F. prausnitzii* and *Gemmiger sp.*, (f) *F. prausnitzii* and *Ruminococcaceae* coassembly, and (g) *Gemmiger sp*. and *Ruminococcaceae* coassembly, generated with Mauve using Locally Collinear Blocks minimum weight of 4000.

We performed extractions of the full-length 16S rRNA gene for these assemblies as well as the full reference genomes for *F. prausnitzii* and partial references for *Gemmiger formicilis* X2-56 and *Gemmiger* sp. An50. The full-length 16S rRNA gene could be extracted for *Gemmiger s*p. An50 but not *Gemmiger formicilis*. We examined the 16S V4 region of these extracted sequences and found they shared 100% identity with the V4 amplicons for the individual cells. Phylogenetic analysis of the full-length 16S rRNA gene sequences revealed that while some single cells clustered with *F. prausnitzii*, others clustered more closely with *G. formicilis* (Figure 6(a)). We mapped our read sets against the *Gemmiger* sp. An50 reference, and found this performed far more poorly than mapping to *F. prausnitzii*, with only 0.0076% to 2.94% percent of reads mapping and only 0.15% to 1.09% of the genome covered. The best coverage of the *Gemmiger* reference was worse than the lowest coverage of *Faecalibacterium* (1.09% versus 1.12%). The poor mapping of many of these single cells to the best available reference genomes suggested that these may represent a novel taxon within the family *Ruminococcaceae*.

Despite the potential novelty of our single-cell genomes, we assessed whether we could extract conserved *Faecalibacterium* genes from our assemblies. We analyzed bile salt hydrolase (BSH), which plays a role in many microbe-host interactions including affecting the efficacy of fecal microbiota transplants,^100^ as well as neuraminidase/sialidase, which cleaves sialic acid and has been implicated in potential interactions between bacteria and viruses in the human body.^99,101^ Again, gene trees for extracted metabolic genes indicated distinct levels of relatedness between single cells not seen in the analysis of full-length 16S rRNA genes. For instance, we noted that 2p1 9H and 2p1 11H group with the other single cells when analyzed by full-length 16S rRNA but encode divergent BSH genes (Figure 6(a,b)). The phylogenetic tree for neuraminidase is less obvious in its topological differences from that of the full-length 16S rRNA gene, but we did observe that 1p5 3B encodes a divergent neuraminidase gene despite tightly clustering with other isolates on the 16S rRNA tree (Figure 6(a,c)). These data once again support our conclusion that high levels of microdiversity are present within single cells that cannot be captured by 16S rRNA gene analysis.

To better characterize the potentially novel *Ruminococcaceae* taxa among our single cells, we performed a co-assembly of the shotgun sequencing reads from the 11 single cells, which were primarily classified as *Gemmiger* by MMSeqs2 (Figure 5(a)). While co-assembly fails to represent the microdiversity inherent in single cells, we sought to leverage this method to further explore this poorly defined taxa. After initial assembly, all contigs below 1500 bp were removed, leaving 456 contigs with a total length of 3,565,808 bps, and a completeness of 98.64% with only 1.52% contamination (Figure 6(d), Supp Table S1). This sequence length compares favorably to *Gemmiger sp*. An50 which has an overall length of 3,241,569 bps. We used Mauve to align *F. prausnitzii* and *Gemmiger sp*. An50 reference genomes to each other, as well as each to our co-assembled *Ruminococcaceae* genome (Figure 6(e-g)), which revealed numerous conserved genomic segments free of internal rearrangements, especially between *F. prausnitzii* and the co-assembled novel *Ruminococcaceae* genome. Average nucleotide identity between *Gemmiger* and *F. prausnitzii* was 78.8%, between *F. prausnitzii* and the co-assembled novel *Ruminococcaceae* was 81.2%, and between *Gemmiger* and the co-assembly was <80%. Thus, we have identified a novel *Ruminococcaceae* taxon that is more genomically similar to *F. prausnitizii* than *Gemmiger*.

### Identification of novel prophages in single-cell genomes

We next sought to identify integrated mobile genetic elements, one of the most variable components of bacterial genomes, in our single-cell sequencing data. Cenote-Taker2 (CT2),^67^ a bioinformatic tool designed for viral discovery and annotation, identified phage-like (*n* = 267) and CTn-like (*n* = 92) elements within our single-cell genome assemblies (Supp Table S3). CT2 was chosen for these analyses as the workflow incorporates both nucleotide- and protein-based searching; compared with other viral discovery software packages, CT2 may reveal fewer overall hits to viral sequences but produces hits with greater substantiating evidence that they are virally derived. *Ruminococcaceae* and *Bifidobacterium* CTns are entirely distinct from one another, sharing no nucleotide similarity, likely the result of the large taxonomic distance between the host genera. Within our *Ruminococcaceae* samples, 80% (24 of 30) of SC genomes contained predicted CTns from one of 4 related groups (Supp Figure S14). In contrast, less than 5% (3 of 62) of *Bifidobacterium* samples carry an apparent CTn, from one related group (Supp Figure S14). These results were unexpected, since at least five CTns are known for *Bifidobacterium* species, while none have been published for *Faecalibacterium* or *Gemmiger*. These results suggest more investigation of *Ruminococcaceae* CTns is warranted, as they may confer relevant phenotypes and contribute to the high levels of genome plasticity already observed within genera from this family.^99^

**Figure 7.**
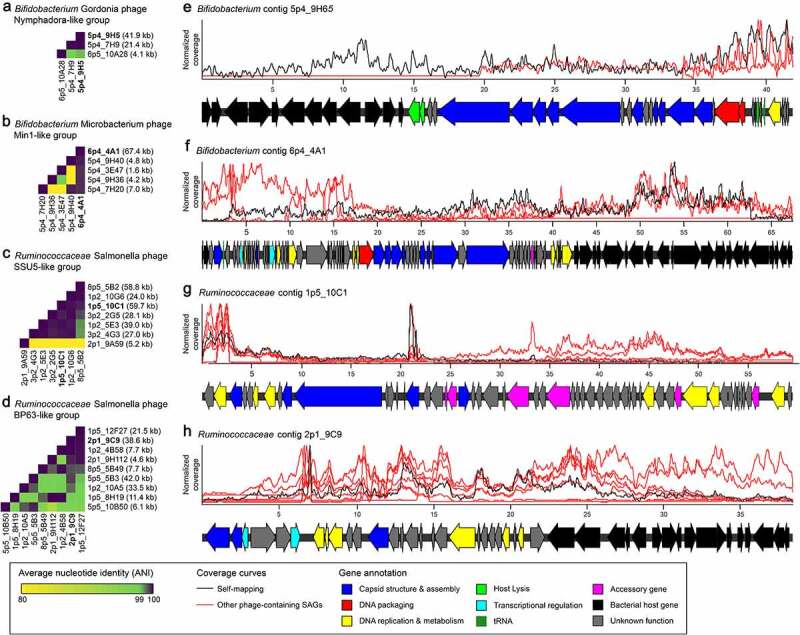
Identification of novel prophage regions integrated in single-cell genomes from human stool. Prophage-containing contigs were defined by their similarity to phage-associated proteins in Cenote-Taker2, and these similarities were used to define related groups. Two groups each are shown from *Bifidobacterium* and *Ruminococcaceae* single-cell genomes. (a-d) Heatmaps display average nucleotide identity (ANI) for all pairs of predicted prophage-containing contigs within a group. The length of each prophage-containing contig is displayed on the right. (e-f) For each group, one representative prophage contig was functionally annotated by a combination of tools (Methods, Supp Table 3). Reads from other single-cell genomes in a group were mapped to the representative and displayed as a coverage curve after normalizing for the total number of reads within a sample. Coordinates along each contig’s length are displayed in kb.

Of the 34 phage groups identified by CT2, 18 were only observed once, suggesting single-cell sequencing is a viable approach to capture a variety of phage types within a sample. Similar to CTns, no overlap in phage groups was observed between *Ruminococcaceae* and *Bifidobacterium* hosts, likely the result of host specificity and the taxonomic distance between these two gut-associated taxa. Four phage groups were chosen for further inspection, two each from *Bifidobacterium* and *Ruminococcaceae* (Figure 7). These phage groups were highly divergent from each other, sharing no significant nucleotide identity (longer than 100 bp) by BLAST.

Each phage group, though defined by a single conserved protein, is highly similar at the nucleotide level (Figure 7(a-d)). A representative contig from each phage group was chosen based on the length of its predicted phage-like sequence for annotation and read mapping from other phage-positive samples (Figure 7(e-h)). Read mapping to these representative contigs agrees with the assemblies of other samples, suggesting that the phage sequence fragments observed are likely due to incomplete sequencing and not gene loss.

Annotation of these contigs identified many phage-associated genes, including those encoding structural proteins, DNA packaging proteins (*e.g*., terminase), and genes for host lysis (*e.g*., holins and lysins) (Figure 7(e-h), Supp Table S3). The combinations of gene functions observed lend confidence to the CT2 phage predictions. Furthermore, gene annotation predicted bacterial host genes in three out of four cases, leading us to conclude that these are likely prophages integrated in the bacterial chromosome, and not the result of phage virions bound to the cell surface during sorting (Figure 5(e-h)). However, chimera formation during amplification, sequencing errors, or mis-assemblies may confound these results. Both *Bifidobacterium* representative phage-containing contigs align to publicly available genomes (Supp Figure S15). The aligned regions only cover the predicted host genes, leading to the conclusion that these are recently acquired integrated prophages.

Finally, the phages identified in these single-cell genomes are entirely novel. Nucleotide BLAST to the RefSeq Viral database did not identify any hits. Likewise, nucleotide BLAST to publicly available host genomes only identified conserved host chromosome regions adjacent to prophages (Supp Figure S15). Taken together, these results highlight the ability of single-cell genome sequencing to discover novel genome diversity, including integrated phages and other mobile genetic elements.

### Novel bacterial genomes can be assembled from rare single cells

Finally, we explored single-cell genomes derived from rarer taxa in our sample set. We chose two sets of cells with at least three representatives each. These cells were classified as *Holdemanella biformis* and *Ruminococcus lactaris* by BLAST search of the 16S rRNA gene V4 amplicon against the RefSeq 16S rRNA sequence database.^88^ We performed assemblies of both these individual cells as well as co-assemblies of the cells within each group. CheckM analysis revealed a completeness between 0.0 and 95.49% (average of 43.35%) for the single-cell assemblies and a completeness of 91.55 and 98.08% for *H. biformis* and *R. lactaris* coassemblies, respectively. Contamination ranged from 0.0 to 13.99% (average of 1.37%) for single cells, with contamination scores of 14.47% and 0.0% for the co-assemblies. The cells used in these analyses, as well as the remaining single cells that were deep-sequenced, exhibited only a 29% correlation with their 16S amplicon-derived assignments by GTDB-Tk analysis.

**Figure 8.**
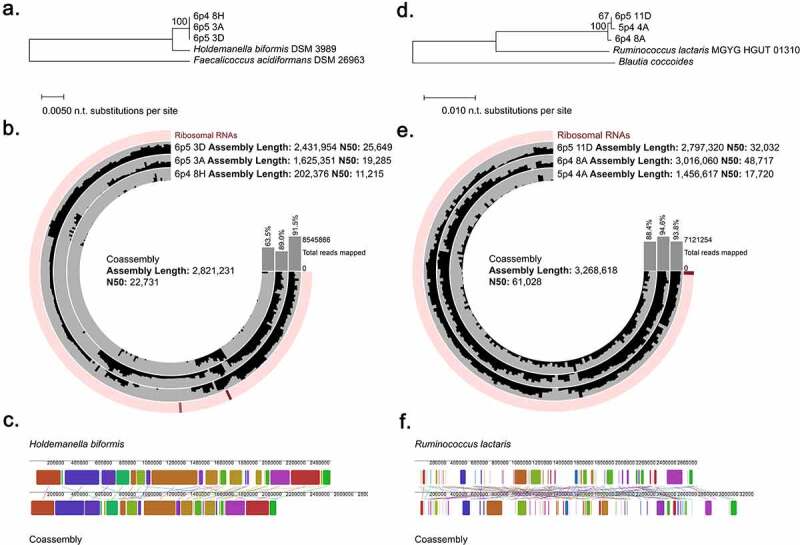
Co-assembly of similar rare microbes improves genomic assemblies and reveals novel taxa. (a) Phylogenetic tree based on full length 16S rRNA genes extracted from single cell assemblies of cells classified as *Holdemanella* by V4 amplicon sequencing. (b) Coverage plot of each individual cell mapped to the co-assembly. Assembly statistics for the co-assembly and individual cell assemblies are displayed as well as the percentage of total reads mapped the assembly. (c) Alignment of co-assembled genome to *Holdemanella biformis* genome. (d) Phylogenetic tree based on full length 16S rRNA genes extracted from single cell assemblies of cells classified as *Ruminococcus lactaris* by V4 amplicon sequencing. (e) Coverage plot of each individual cell mapped to the coassembly. Assembly statistics for the co-assembly and individual cell assemblies are displayed as well as the percentage of total reads mapped the assembly. (f) Alignment of co-assembled genome to *Ruminococcus lactaris* genome.

We first examined the cells most closely related to *H. biformis*. Examination of full length 16S rRNA gene sequences revealed that the single cells all clustered more closely together than they did to the reference (Figure 8(a)), supporting coassembly to resolve a potentially novel genome. The coassembly performed better than individual single-cell assemblies, with an increase in total assembly length of 2,821,231 base pairs compared to the average length for single-cell assemblies at 1,453,227 base pairs. The coassembly length compares favorably to the *H. biformis* reference genome which has a length of 2,415,920 base pairs (Figure 8(b)). We next mapped individual reads to the co-assembly and found all read files contributed to the assembly (Figure 8(b)). Alignment of the *H. biformis* reference genome and our co-assembly revealed numerous conserved segments (Figure 8(c)), but overall average nucleotide identity was 89.9%, suggesting our co-assembled genome is likely to represent a novel species of *Holdemanella*.

We performed similar analyses on the cells classified by 16S rRNA gene V4 analysis as *Ruminococcus lactaris*. These single cells also clustered more closely together than to the reference genome but were more divergent than what we observed for the *Holdemanella* single-cell set (Figure 8(d)). Again, the co-assembly was improved in both length and N50 value in comparison to individual cell assemblies, and there was a more equitable contribution of coverage from all cells as compared to the *Holdemanella* cell set (Figure 8(e)). The co-assembly had an overall genome length of 3,268,618 base pairs, which compares well to the reference genome which had a length of 2,731,236 base pairs. Alignment of the *R. lactaris* reference genome and our co-assembly revealed conserved segments (Figure 8(f)) but overall average nucleotide identity was <80%, indicating that we have identified a new species of *Ruminococcus* or possibly a new genus in the family *Ruminococcaceae.*

These analyses present clear evidence that co-assembly of similar single cells can improve the overall representation of the target genome. It is also important to note that the closest reference as defined by 16S rRNA gene V4 amplicon may not reflect the true taxonomic lineage of these cells.

## Discussion

Here, we describe the application of FACS-based isolation and MDA of single bacterial cells from complex microbial communities in human fecal samples, followed by careful screening of single cells for shotgun-sequencing and resolution of genomic features. Approaches to the delineation of bacterial genomes from complex samples are still evolving to overcome numerous obstacles related to difficult-to-cultivate and poorly characterized taxa, clear linkage of specific genetic factors to the genome of origin, and resolution of the diversity of genetic features present in a single species within individual samples. In addition, high-resolution sequencing and bioinformatic analysis of metagenomes can be prohibitively expensive. The method presented here offers numerous practical advantages and facilitates selective application of resources toward desired cells.

Our approach offers two potential opportunities for informed selection of cells to prioritize for shotgun-sequencing. Individual wells with non-optimal SYBR Green MDA profiles could be excluded or de-prioritized even for 16S rRNA gene PCR and sequencing, as we observe a consistent correlation between non-sigmoidal or late-amplifying curves with failure to yield a band after 16S rRNA gene PCR (Figures 1(b) and 2(d)). A second, and more critical, selection can be performed after 16S rRNA gene amplicon analysis, wherein likely contaminants can be readily removed and/or only cells corresponding to targeted taxa of interest to the investigator can be specifically selected. Here, we prioritized *Bifidobacterium, Faecalibacterium*, and several rarer taxa for further analysis, but suggest that this approach offers enormous flexibility in terms of allocating resources toward high-yield targets. As has been reported using numerous other approaches for the study of microbiota samples, the “kit-ome” is a major consideration here, especially based upon the sensitivity of this method for amplifying single bacterial cells. Presumed contaminants represented approximately 50–60% of our single cells despite rigorous UV treatment of plates and reagents and consistent use of a PCR hood for all procedures, emphasizing the importance of a stringent bioinformatic filtering pipeline to minimize a potential waste of resources in sequencing these interlopers.

With regard to optimizing recovery of desired taxa, several factors may be worth considering during experimental design. While our method yields representative members of a wide variety of taxa, we observed a consistent bias in our recovery of *Bifidobacterium* at the cost of other community members, likely secondary to the differential fluorescence intensity of SYTO-9 for different bacterial taxa.^86^ Gentle alkali lysis^102^ may also introduce bias, either through failure to lyse cells or alternately degradation of DNA of some species. Our use of detectable 16S rRNA gene(s) by PCR as a selection marker may also bias recovery towards genomes with 16S rRNA genes that are more readily accessible to Phi29.^103^ Finally, it is known that abundance profiles from amplicon sequencing may not fully reflect the true abundance of taxa in a sample,^104^ making it challenging to even evaluate bias in single cell recovery. While completely unbiased sorting of bacterial cells from complex communities may prove intractable, these potential sources of bias may also represent opportunities for enrichment of specific taxa of interest. For example, bacteria that stain less well with nucleic acid stains may benefit from selection of the lower 50% of stain-positive bacteria for sorting, while for others, more aggressive lysis methods may be indicated. Finally, targeted enrichment using semi-selective dyes, antibodies, or probes, if available, could be applied to maximize the efficient recovery of specific taxa.

Here, we have demonstrated that shotgun sequencing of individual bacterial cells is a powerful tool for characterizing multiple genomic factors, both intimately linking genetic features to the host cell of origin and revealing microdiversity within a sample. Our analyses using *E. coli* indicate that we are often able to capture nearly complete genomes from single-cells, though coverage is variable (Figure 1(c)). While less practical to determine the genomic coverage from microbiota-derived single cells which are unlikely to perfectly match reference genomes, our explorations of groups of single cells from matched genera indicate that we similarly achieve variable, but often robust, coverage of these genomes (Figures 3(a) and 5(a)). We elected to analyze a well-characterized genus with numerous available reference genomes (*Bifidobacterium*), as well as a genus that has been implicated in numerous disease states^105,106^ but for which limited reference genomes are available (*Faecalibacterium*). For both bacterial types examined, the genomic diversity observed through metabolic marker gene analysis was greater than what could be resolved even by full-length 16S rRNA gene analysis alone. Importantly, we have only scratched the surface of the potential genetic insights available from these cells. While function can be suggested by taxonomy, access to primary sequencing data from individual cells provides direct evidence toward the functional capacity of specific microbes. Data from these single cells have the potential to reveal previously unrecognized diversity of known genes as well as novel genes, especially given that genes of unknown function account for almost 70% of gut microbiome genes.^107–109^

*Bifidobacterium* is the predominant symbiont in the breastfed infant microbiome,^110,111^ and in school-aged children and adults, levels are inversely correlated with many disease states including obesity,^112^ ulcerative colitis,^113^ and *Clostridium difficile* infection.^114^ Prior studies have indicated that *Bifidobacteria* inhibit inflammation through unknown secreted factors,^115,116^ and limit invasion of pathogens by altering gut lumen pH and distribution of short-chain fatty acids.^117^ Metagenomic studies of the infant microbiome have also been used to explore the presence or absence of *Bifidobacterium-*derived enzymes to metabolize human milk oligosaccharides.^118^ Our analysis of *Bifidobacterium* single cells from an adult individual indicates enormous complexity of this genus, with representation of numerous described species, but also presence of taxa divergent at even the full-length 16S rRNA gene level with genes more closely related to other species (Figure 4). Improved understanding of how *Bifidobacterium* benefits the human host will require knowledge of its entire genome, not just its relative abundance, to identify genes of interest and elucidate mechanisms.

Few strains of *Faecalibacterium* have been sequenced, suggesting that there is likely a high degree of unexplored genomic diversity within this genus.^119^ Relative abundance of *Faecalibacterium* species has been inversely correlated with many disease states, including inflammatory bowel disease, colorectal cancer, and diabetes,^106,120^ and this genus has the capacity to produce anti-inflammatory metabolites including microbial anti-inflammatory molecule,^121^ butyrate,^122^ and extracellular polymeric matrix.^123^ However, not all strains produce the same types of these metabolites, highlighting the importance of strain-level differences. Our analysis of *Faecalibacterium* genomes revealed poor alignment to deposited reference genomes; this observation is consistent with prior reports indicating that 16S rRNA gene-based assignments may be especially inaccurate for *Faecalibacterium* because of its incredible genome plasticity, likely the result of horizontal gene transfer.^99^ Our delineation of several novel genome assemblies from our single cell genomes emphasizes the utility of these data beyond analysis of conserved pathways; they can be used to better define the full genomic landscape of taxa for which related reference genomes are lacking. Combining genomic information from 11 cells defined as *Ruminococcaceae* by 16S rRNA gene analysis yielded an assembly with length 3,565,808 base pairs and an N50 of 12,562. This is a large improvement over the individual assembly lengths which were an average of 1,615,064 base pairs. We applied this even more directly in our analysis of several groups of cells with ASVs that only exhibited 95–96% average nucleotide identity to reference databases by 16S rRNA gene analysis (*Holdemanella* and *Ruminococcus*). Individual cell assemblies generated genomes with an average length of 1,453,227 and 2,423,332 base pairs and an N50 of 18,716 and 32,823 base pairs respectively. Merging together reads from cells with identical 16S rRNA gene V4 amplicons improved assembly length to 2,8231,231 and 3,268,618 and N50 values to 22,731 and 61,028, respectively.

Perhaps one of the greatest challenges for analysis of metagenomic data from complex communities is the assignment of mobile genetic elements like CTns and prophages to bacterial host genomes of origin. Understanding of strain-level diversity requires an examination of these elements, which are transferred between microbes in the gut at high rates.^17^ Integrative mobile genetic elements like CTns and temperate phages are known to carry a diverse set of genes beneficial to their hosts, since their fitness becomes inextricably linked to that of the host upon integration. CTns have been implicated as a primary reservoir for antibiotic resistance genes in the human gut,^124,125^ and we report multiple clusters of CTns among our single cells. Our identification of novel *Ruminococcaceae* CTns are of particular interest, as none have been previously reported for either *Faecalibacterium* or *Gemmiger*. Temperate phages can carry virulence factors^126^ and metabolic enzymes^127,128^ and transfer those functions horizontally between contemporary bacterial hosts. While beneficial mobile genetic elements have been widely studied in pathogenic hosts (e.g. toxin-producing phage),^19^ the impact of these elements on commensal bacteria has been underexplored despite these likely being key to determining how the gut microbiota functions in health and disease.^129^ Temperate phages are especially abundant^130^ and diverse^131^ in the gut, suggesting they play a role in maintenance and remodeling of the gut microbiota. These were readily detectable in our single-cell genomes, indicating the utility of single-cell genome analysis for efficiently and accurately identifying phage-host pairs. Culture-based approaches to phage discovery are highly biased by the availability of cultivable hosts, culture conditions, and the appearance of plaques. Analysis of the virome often fails to sample diversity deeply enough^131^ and can be biased by the chosen assembly method.^132^ Additionally, it is often difficult to predict susceptible bacterial hosts.^133^ A recently described method for single cell viral tagging of phage bound to bacteria identifies surface-level interactions but does not ensure bacterial hosts are susceptible or that phages are infectious.^40^ Our identification of numerous novel prophages in *Bifidobacterium* and *Ruminococcaceae* genomes (Figure 7) emphasizes the opportunities for complementary single-cell analyses of tagged and untagged cells to delineate phage–host interactions.

In conclusion, our method using FACS for single-cell sorting of bacteria derived from human stool, whole-genome sequencing of those single cells, and bioinformatic analysis yielded genomic data for a variety of different microbiota-derived single cells. We present a means to readily prioritize sample-derived taxa of interest for deep-sequencing using 16S rRNA gene analysis, and report that we are able to capture metabolic genes, CTns, and prophages. A full understanding of the gut microbiome requires finer resolution of bacterial genomes than what marker- and metagenomic-based approaches can offer, and single-cell genomic resolution offers a powerful method to definitively link a variety of conserved and mobile genomic features with 16S rRNA gene-based taxonomic assignments and to reveal the microdiversity present among the complex communities in the human microbiota.

## Supplementary Material

Supplemental Material

## Data Availability

All sequencing data generated and analyzed in this study are available at NCBI BioProject ID PRJNA733504 (https://www.ncbi.nlm.nih.gov/bioproject/PRJNA733504). Custom scripts have also been made available.^44^
