# Supplementary material for: Single-cell genomics for resolution of conserved bacterial genes and mobile genetic elements of the human intestinal microbiota using flow cytometry
Source: Gut Microbes. 2022 Feb 7;14(1):2029673. doi: 10.1080/19490976.2022.2029673 (PMC8824198; doi:10.1080/19490976.2022.2029673)
Supplement: Supplemental Material [file KGMI_A_2029673_SM1460.zip › supplementary/211122_SCG_Supplement_Fig_resubmit_final.docx]

**Supplemental Figures**

**
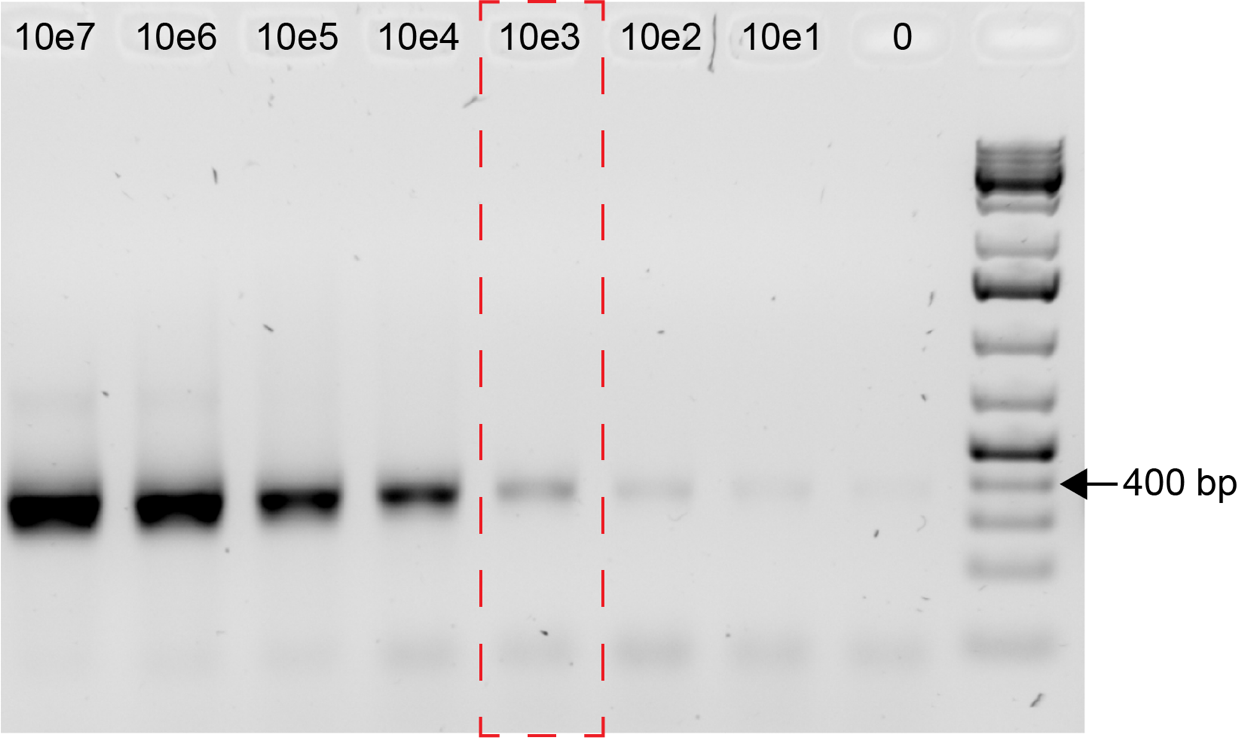
**

**Supplemental Figure 1. Limit of detection for 16S rRNA gene V4 amplicon PCR reaction is 10e3 copies.** 16S rRNA gene V4 PCR was performed using known copy numbers of a plasmid containing a V4 insert, which has previously served as a standard for RT-qPCR analyses. Image is representative of three independent replicates. Limit of detection for PCRs to be considered “positive” is shown in outlined box.


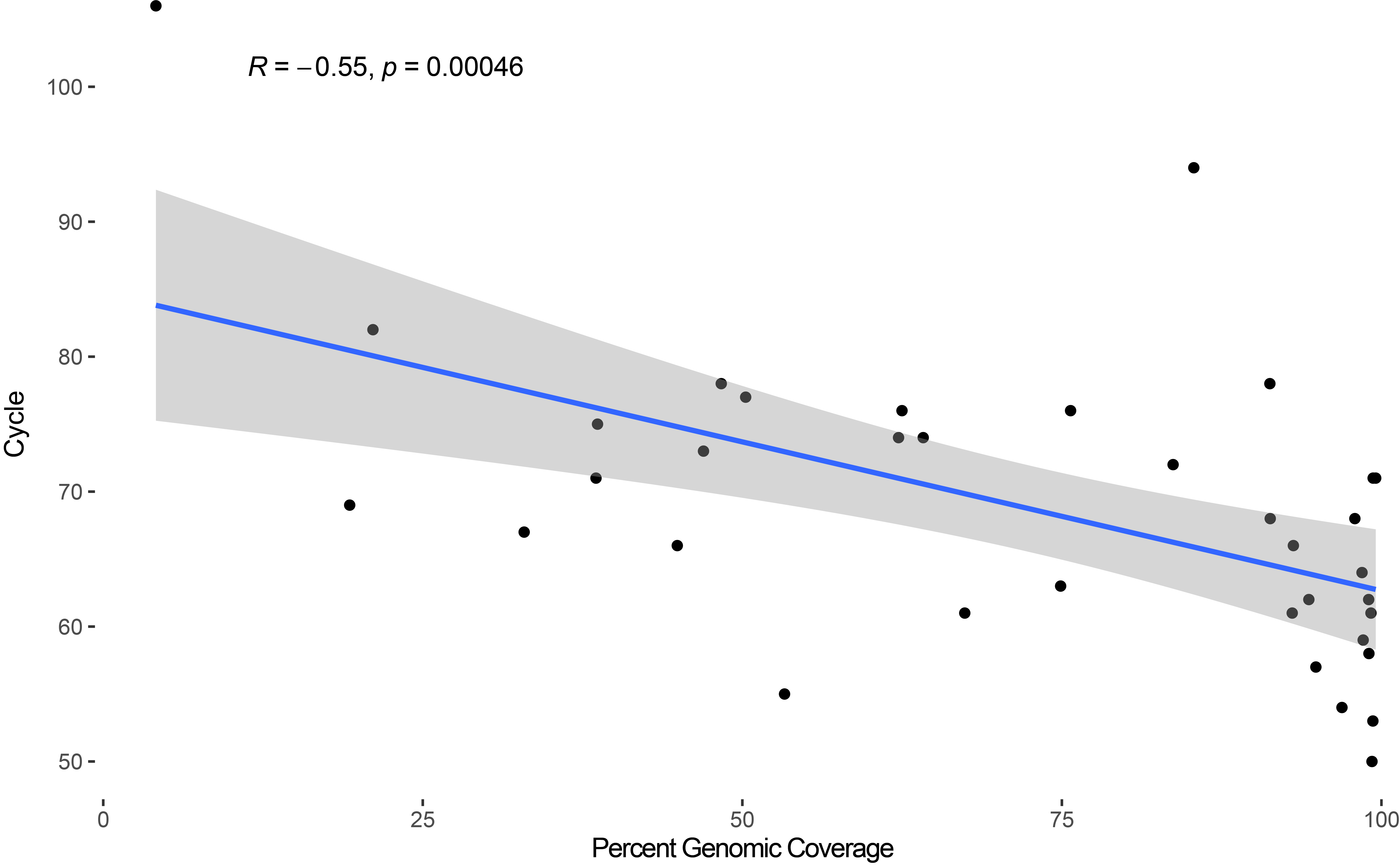


**Supplemental Figure 2. Correlation between genomic coverage and inflection point of SYBR Green amplification curve.** Inflection points of the SYBR Green multiple displacement amplification curves were defined as the first cycle with a normalized value greater than or equal to 50% maximal fluorescence. The inflection points were then compared to the genomic coverage for individual *E. coli* single cells when mapped to the reference genome. Curve was fitted using *lm* and Pearson correlation coefficient was generated using *stat_cor*.

**
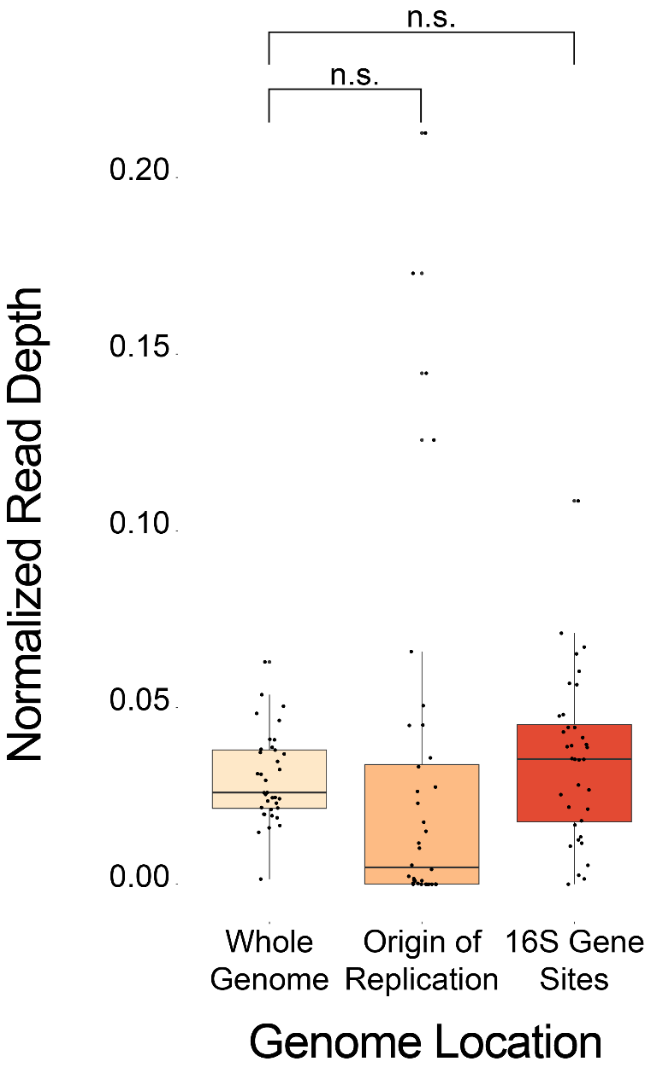
**

**Supplemental Figure 3. The origin of replication and 16S rRNA genes do not represent regions of increased read depth compared to the remainder of the *E. coli* genome.** Comparison of read depths normalized to the maximum depth at any site within each cell for the indicated regions was performed by ANOVA and Tukey’s test. Origin of replication and 16S rRNA gene were extracted by OriFinder and barrnap, respectively.


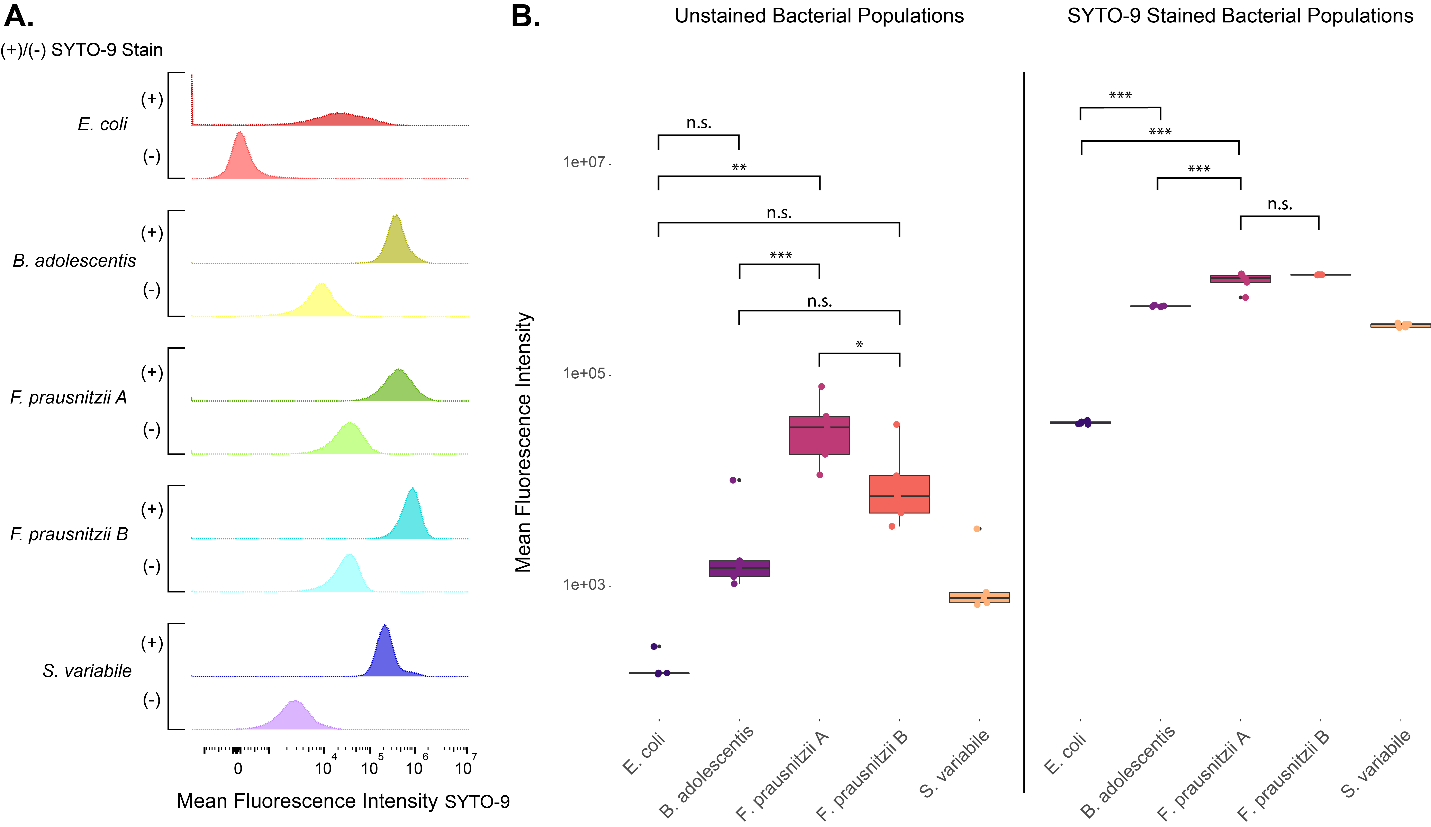


**Supplemental Figure 4. *B. adolescentis* and *F. prausnitzii* isolates exhibit significantly greater SYTO-9 fluorescence compared to *E. coli*. (A)** Mean fluorescence intensity of the indicated bacterial isolates with or without SYTO-9 staining was assessed using flow cytometry. **(B)** Mean fluorescence intensity was analyzed by one-way ANOVA and post-hoc Tukey’s Test (n=3).


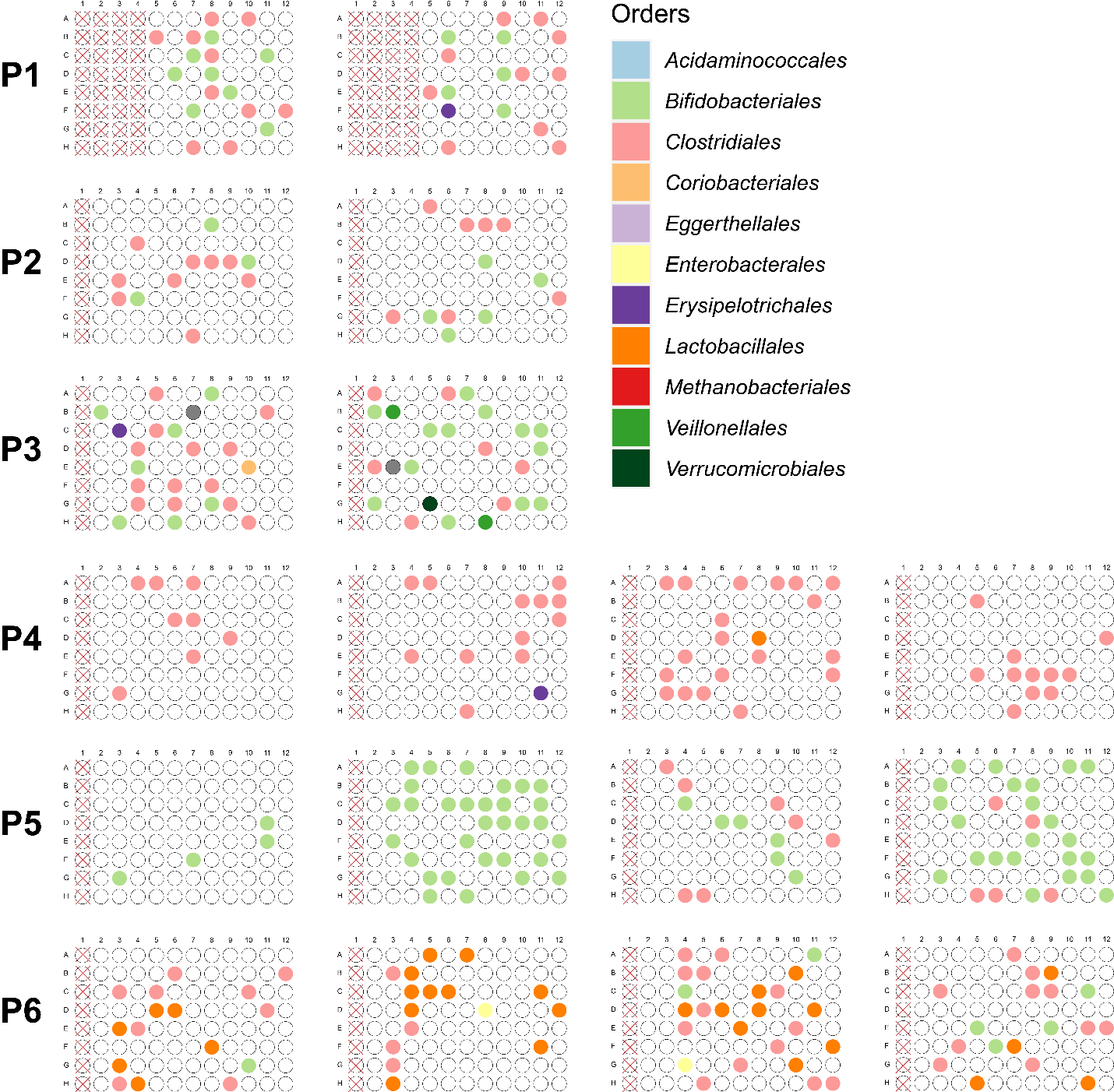


**Supplemental Figure 5. Original sort location of individual cells in 96-well plates.** Plates are arranged by sample of origin. Samples were sorted by row. Empty wells represent either 16S-negative wells or 16S-positive wells that failed filtering. Wells marked with a red “X” did not receive a sorted cell.

**
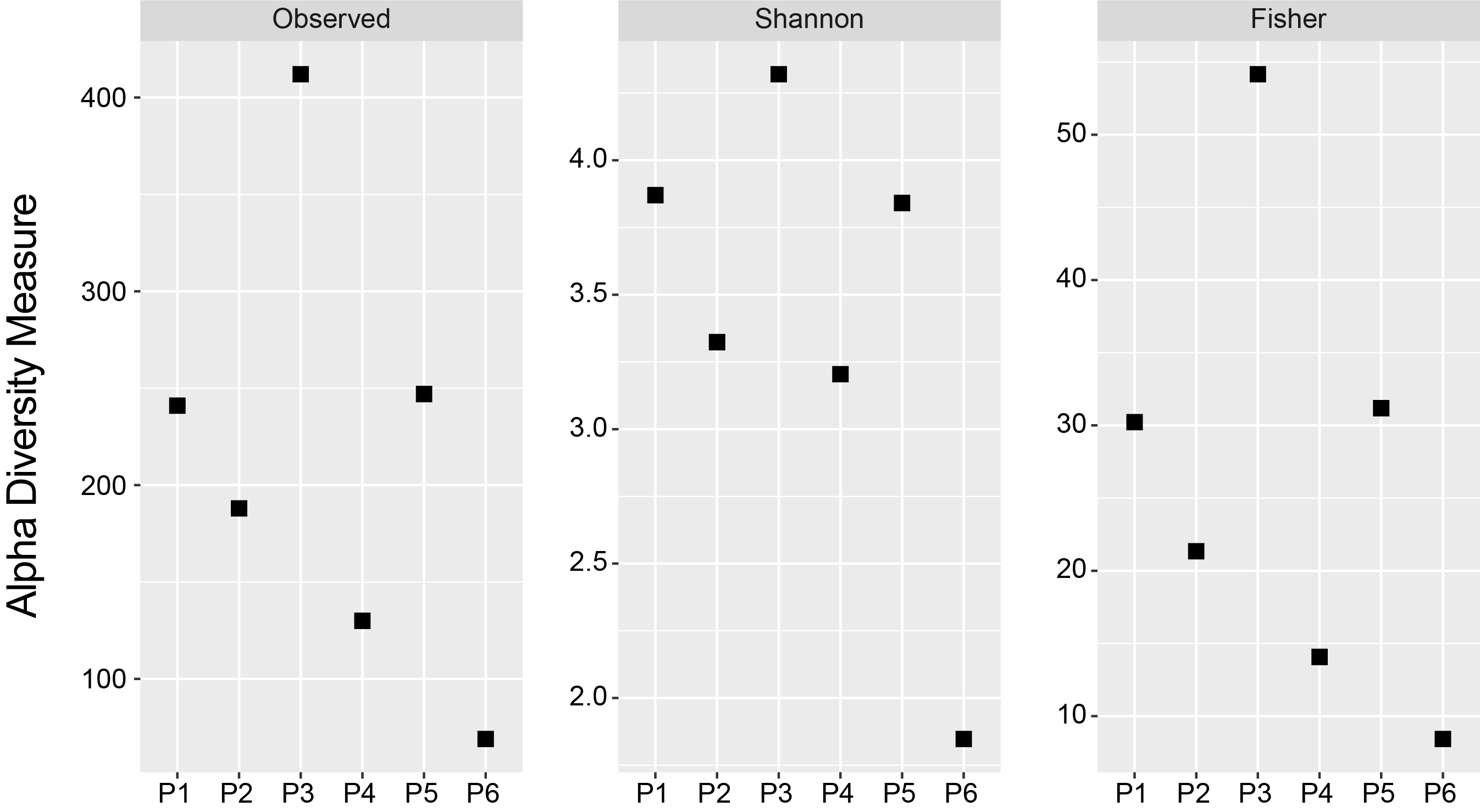
**

**Supplemental Figure 6. Alpha diversity analysis reveals Patient 3 has the highest diversity among six samples analyzed.** Observed species, Shannon diversity and Fisher’s alpha were determined for the 16S rRNA gene V4 amplicon sequencing results for P1-P6 communities.

**
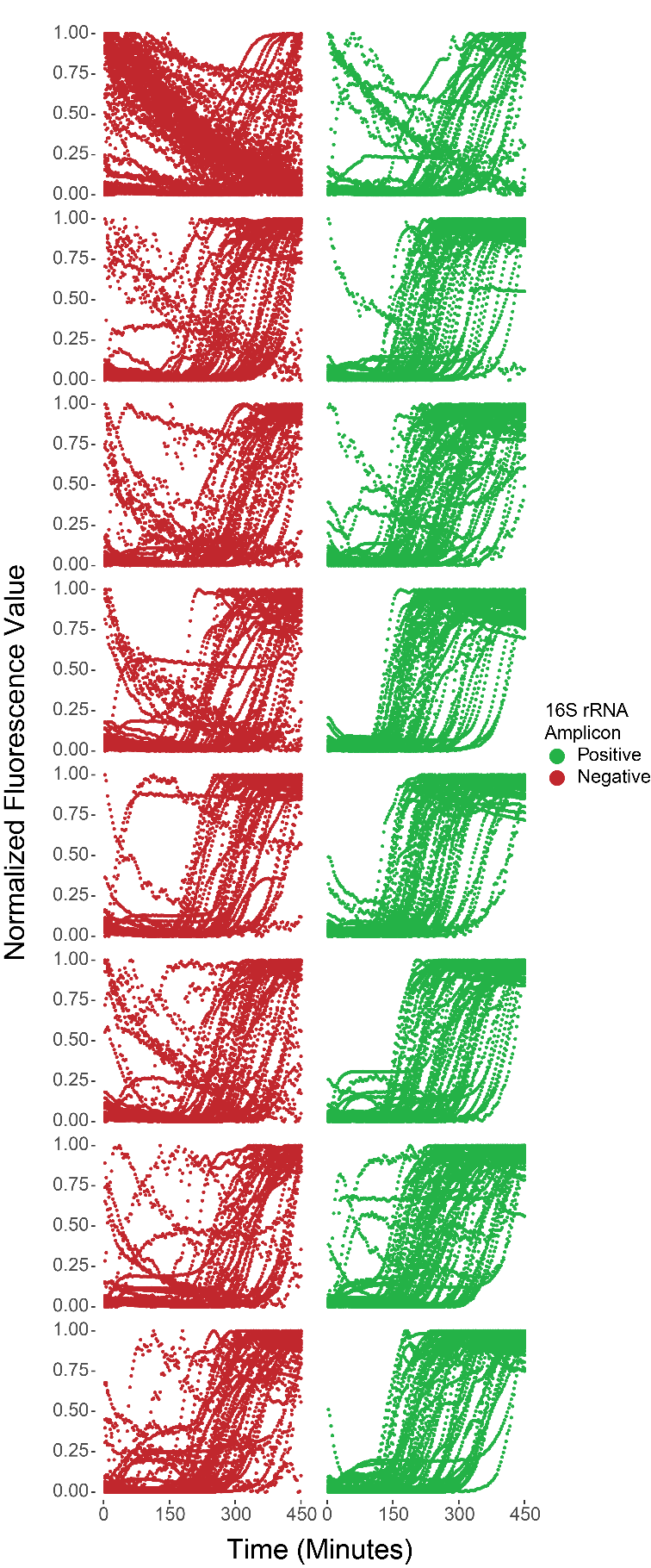
**

**Supplemental Figure 7. SYBR Green curves generated from MDA of single cells sorted from Patient 3.** All curves represent normalized multi-component data collected once every three minutes. Samples were organized by 16S rRNA V4 amplicon presence detected *post hoc*.

**
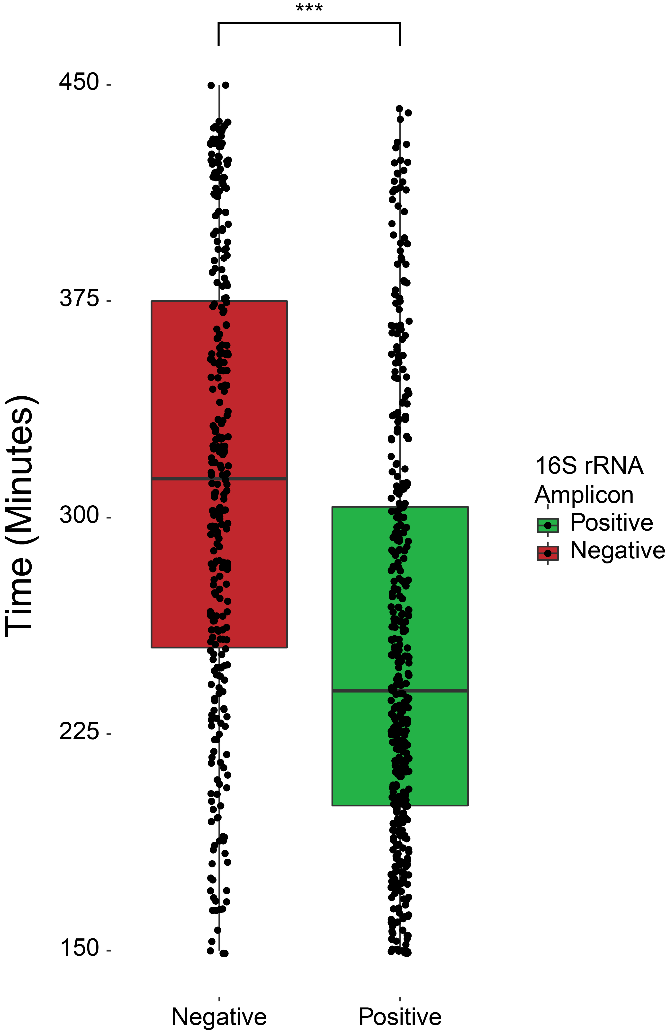
**

**Supplemental Figure 8. 16S-positive samples had significantly earlier inflection points in their sigmoidal curves.** Inflection points on curves were defined as the first value with a normalized value greater than or equal to 50% maximal fluorescence. Analyzed by Mann-Whitney test with P< 2e-16.

**
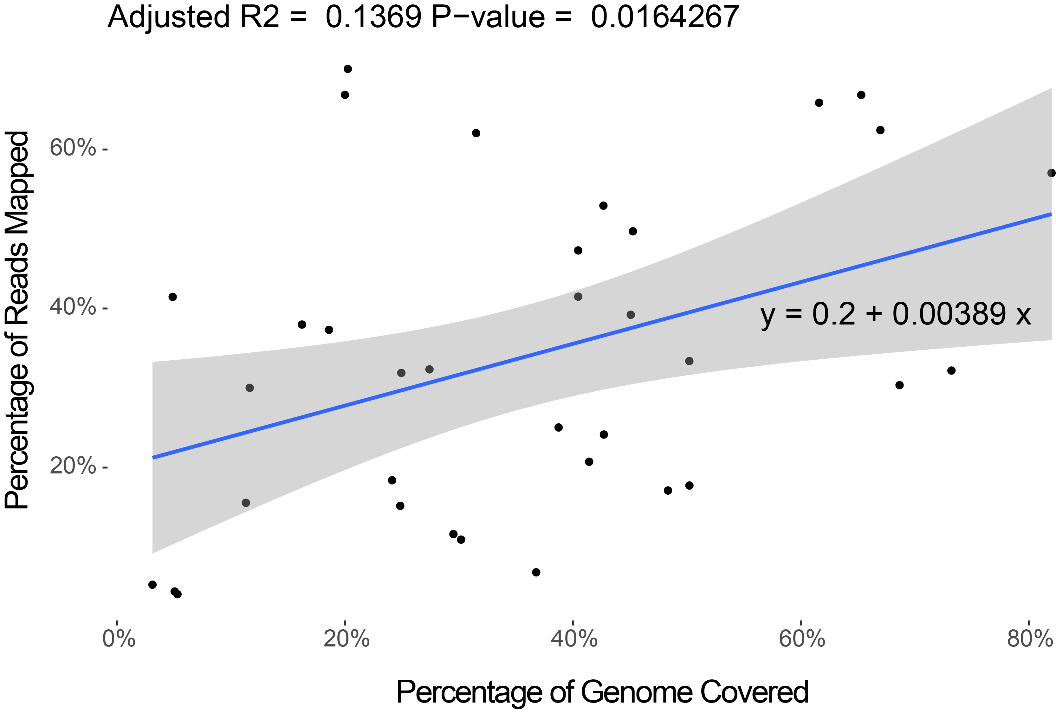
**

**Supplemental Figure 9. The percentage of reads mapped correlates with percentage of genome covered in analysis of microbiota-derived *Bifidobacterium* single cells.** Curves were fitted with a standard linear mixed model in R using the lm function.

**
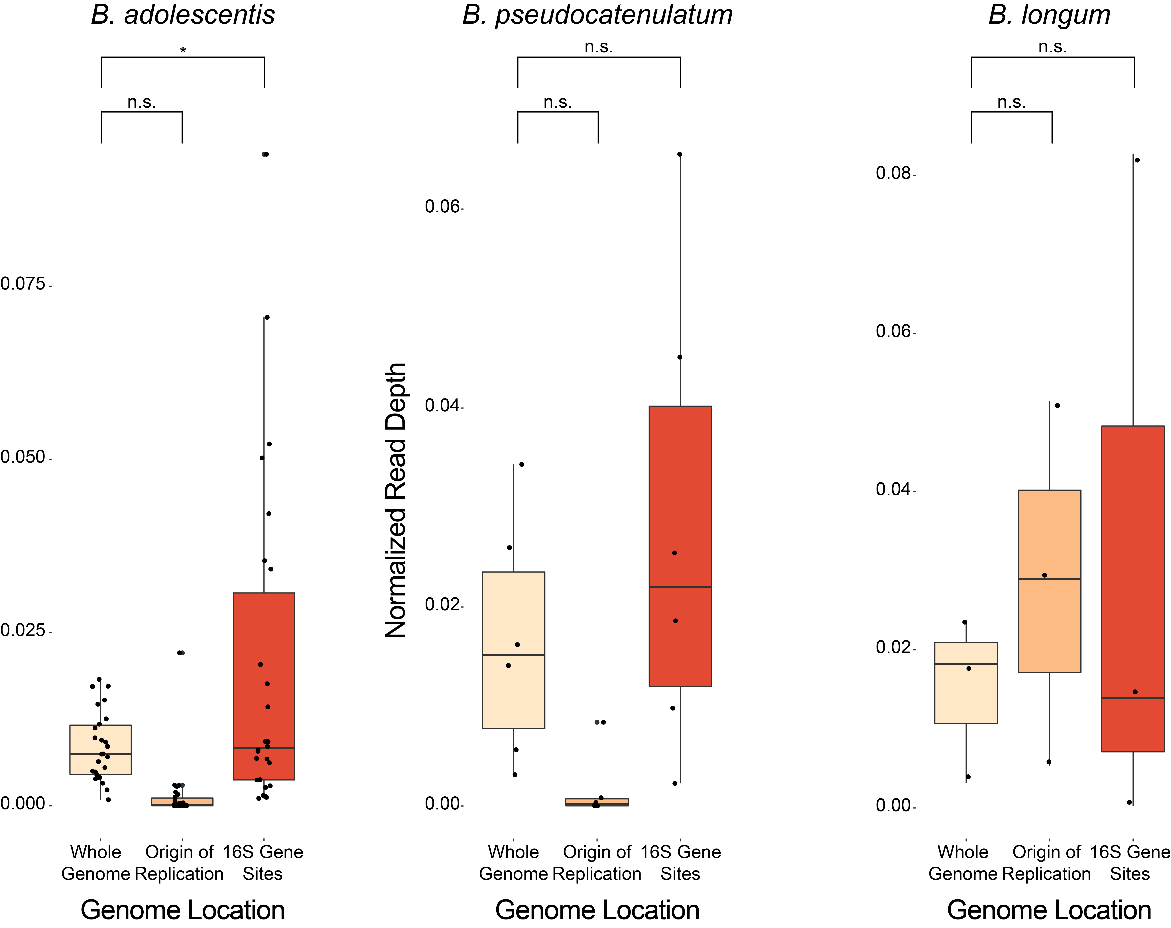
**

**Supplemental Figure 10. 16S rRNA genes recruit *Bifidobacterium adolescentis* single cell reads to a greater degree than the remainder of the genome.** Comparison of normalized read depth for the indicated regions was performed using ANOVA and Tukey’s test. Origin of replication and 16S rRNA gene sites were extracted by OriFinder and barrnap, respectively.


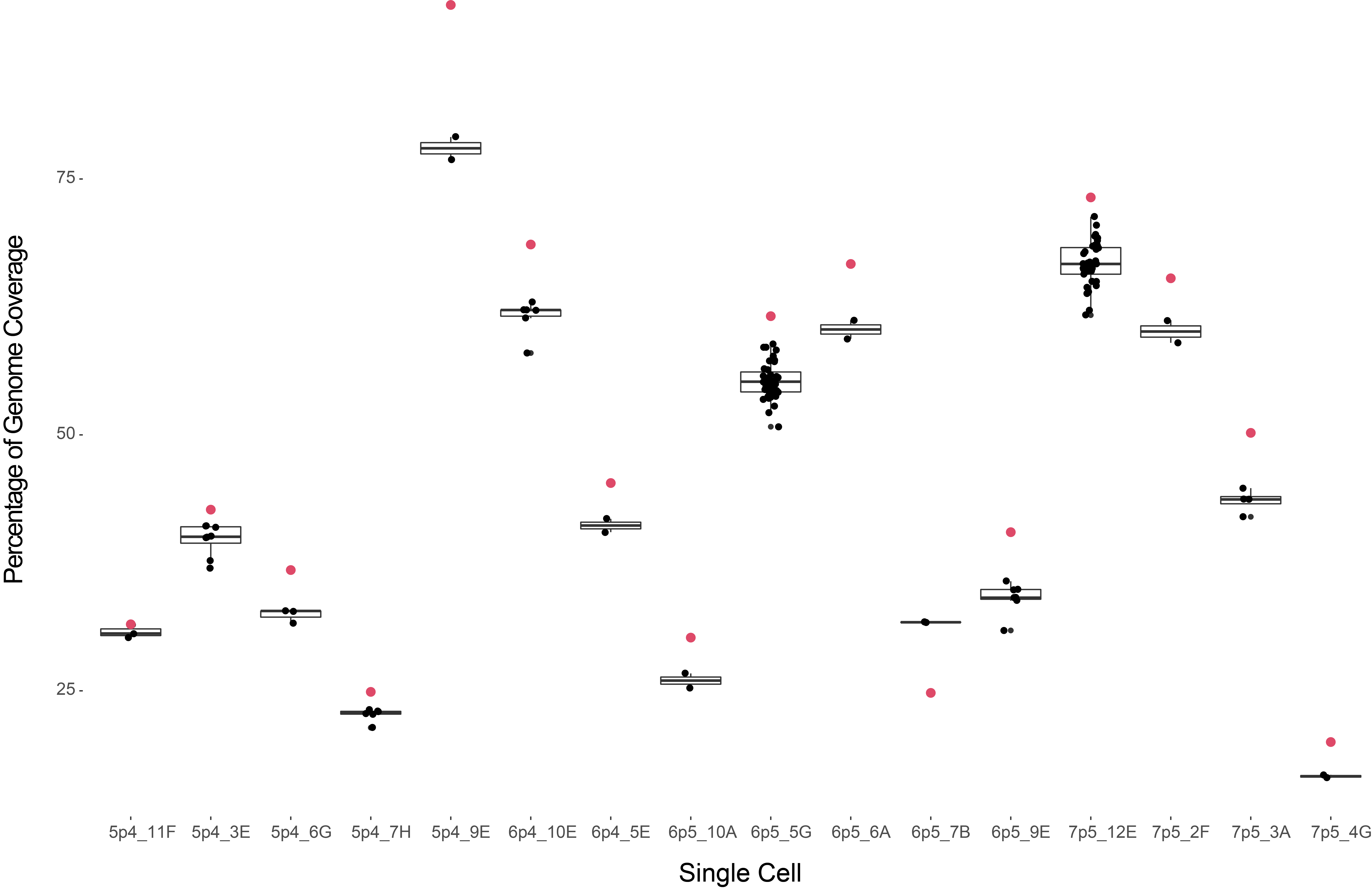


**Supplemental Figure 11. Mapping of single cell reads to reference genomes selected by ReferenceSeeker is similar to mapping to the reference selected by 16S rRNA for *Bifidobacterium* cells.** All reads were mapped using bowtie2 against reference genomes selected by ReferenceSeeker (black dots) or by 16S rRNA (red dots).

**
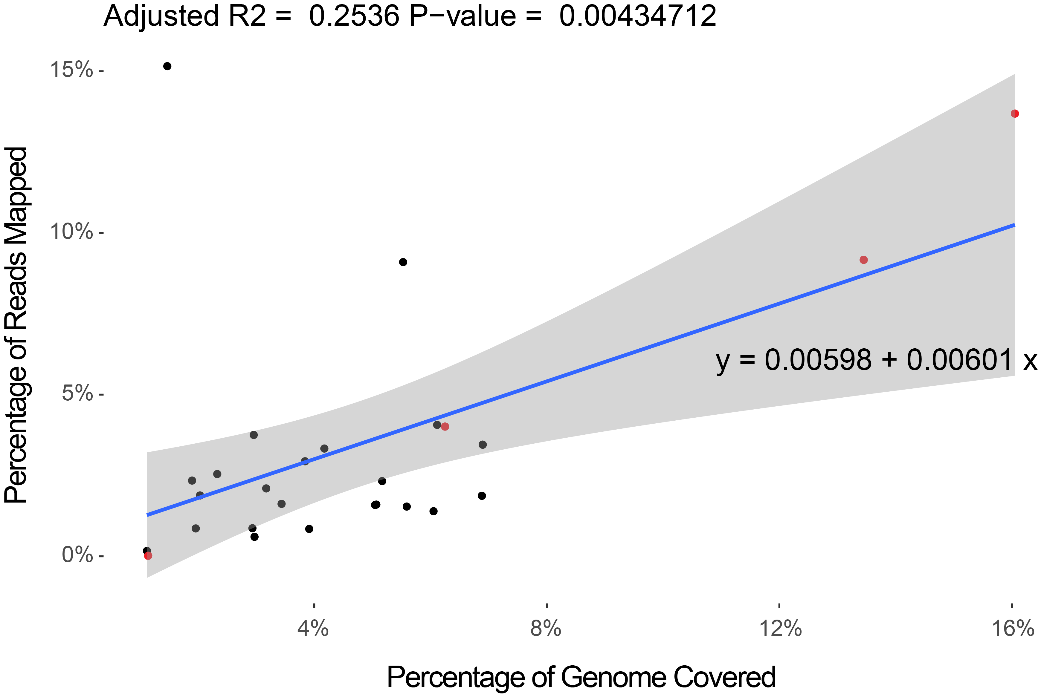
**

**Supplemental Figure 12. The percentage of reads mapped correlates with percentage of genome covered in analysis of microbiota-derived *Faecalibacterium* single cells.** Curves were fitted with a standard linear mixed model in R using the lm function. Samples in red (n = 4) are those most closely related to *F. prausnitzii* by full-length 16S rRNA gene comparison.

**
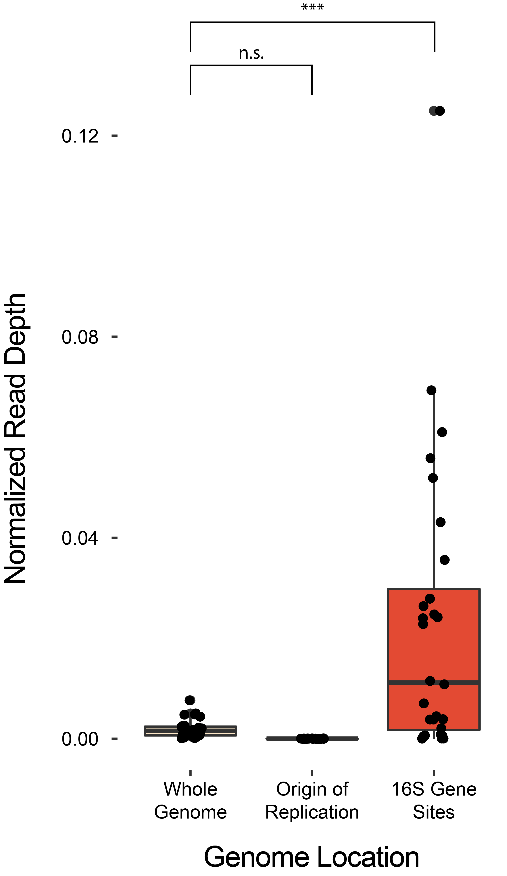
**

**Supplemental Figure 13. 16S rRNA genes recruit *Faecalibacterium* single cell reads to a greater degree than the remainder of the genome.** Comparison of normalized read depth for the indicated regions was performed using ANOVA and Tukey’s test. Origin of replication and 16S rRNA gene sites were extracted by OriFinder and barrnap, respectively.

**
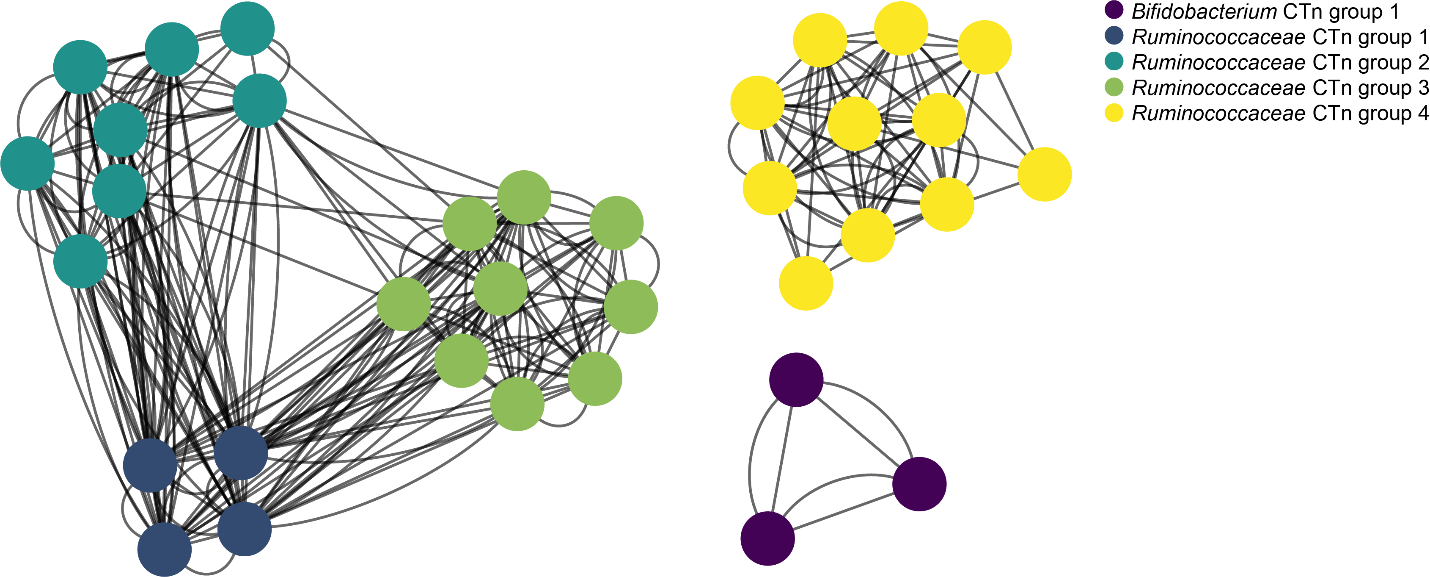
**

**Supplemental Figure 14. Network analysis of predicted CTn-containing contigs.** Nodes represent CTns and nucleotide similarity between *Ruminococcaceae* and *Bifidobacterium* CTns are represented by network edges. Nucleotide similarity was calculated as percent length aligned from reciprocal nucleotide BLAST. Node colors represent CTn clusters, which were defined by MCL with an inflation parameter of 2. Network was visualized with Cytoscape.

**
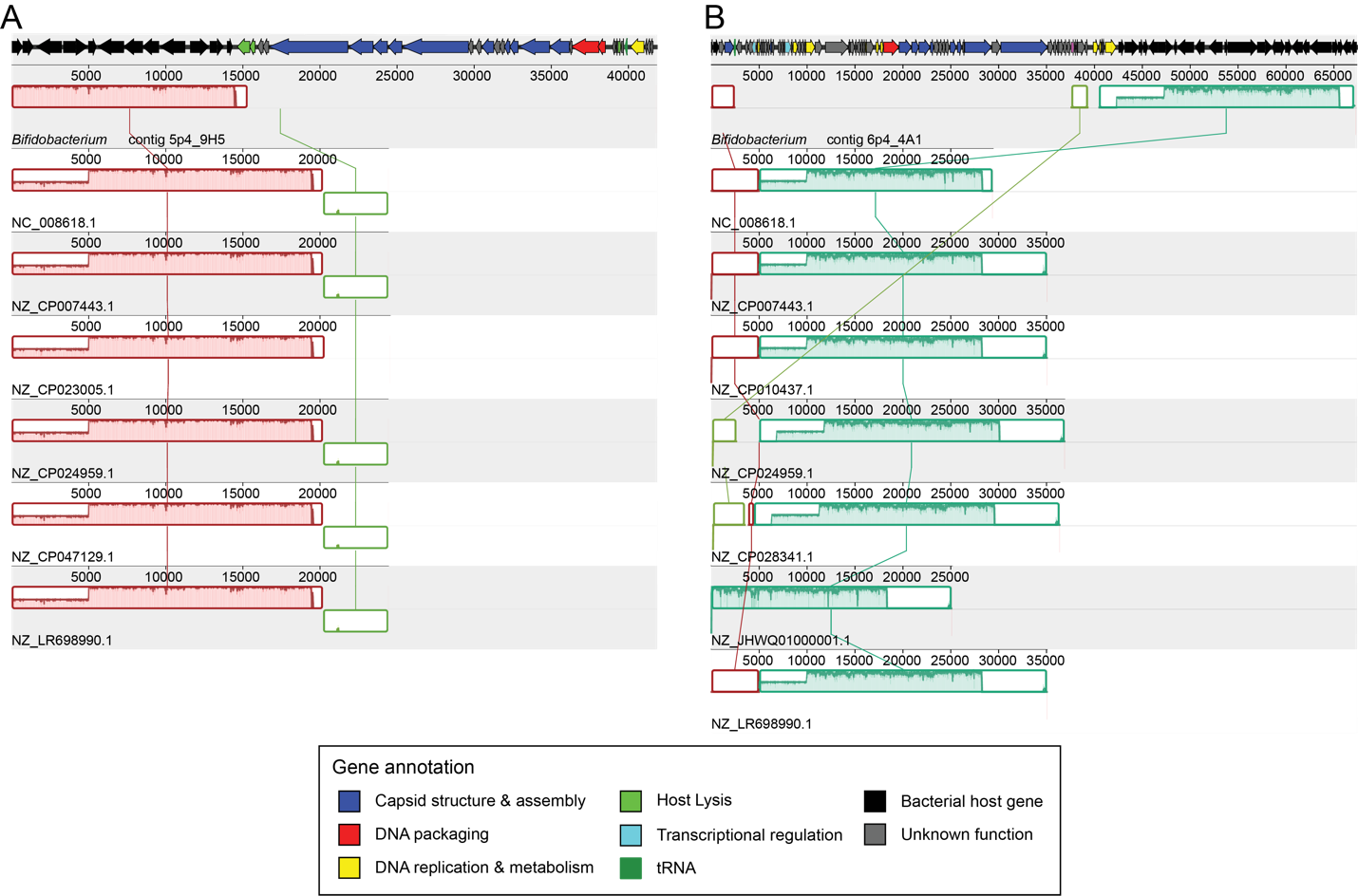
**

**Supplemental Figure 15. *Bifidobacterium* prophage-containing contigs contain host chromosome sequence that aligns to publicly-available genome assemblies. (A)** *Bifidobacterium* contig 5p4_9H5 is the representative contig for the group of Gordonia phage Nymphadora-like prophages, and aligns to six publicly-available *B. adolescentis* genomes. **(B)** *Bifidobacterium* contig 6p4_4A1 is the representative contig for the group of *Microbacterium* phage Min1-like prophages, and aligns to six publicly-available *B. adolescentis* genomes and one *B. ruminantium* genome (NZ_JHWQ01000001.1). Gene annotations for each representative prophage-containing contig are as in **Fig 7**. Related regions from publicly available genome assemblies include 5 kb of flanking sequence on either side of the homologous region identified. Genomes are referred to by their NCBI accession numbers. Coordinates along the length of each contig and genome region are displayed in bp.
