# Supplementary material for: Single-cell genomics for resolution of conserved bacterial genes and mobile genetic elements of the human intestinal microbiota using flow cytometry
Source: Gut Microbes. 2022 Feb 7;14(1):2029673. doi: 10.1080/19490976.2022.2029673 (PMC8824198; doi:10.1080/19490976.2022.2029673)
Supplement: Supplemental Material [file KGMI_A_2029673_SM1460.zip › supplementary/Bifidobacterium_MMSeqs_Report.html]

Uploaded\_sample\_set-report.utf8.md


# Classification report for Uploaded sample set

#### Pavian R package v0.8.4

#### Fri May 28 20:31:00 2021

# Sample set summary

- Classification summary
- Raw read numbers
- Sample information

# Classification results

- Bacteria
- Viruses
- Eukaryotes
- Eukaryotes/Fungi
- Eukaryotes/Protists

Showing 100 of 3369 species.

# Sankey visualization

## 5p4\_11F

## 5p4\_3E

## 5p4\_5A

## 5p4\_5C

## 5p4\_6B

## 5p4\_6G

## 5p4\_7E

## 5p4\_7H

## 5p4\_9E

## 5p4\_9H

## 6p4\_10E

## 6p4\_10G

## 6p4\_12A

## 6p4\_4A

## 6p4\_4H

## 6p4\_5E

## 6p4\_5F

## 6p5\_10A

## 6p5\_12E

## 6p5\_3H

## 6p5\_5G

## 6p5\_6A

## 6p5\_7B

## 6p5\_7C

## 6p5\_7E

## 6p5\_9E

## 7p5\_10F

## 7p5\_11D

## 7p5\_11E

## 7p5\_12E

## 7p5\_2F

## 7p5\_3A

## 7p5\_4G

## 7p5\_5H

## 7p5\_8F

# About

This file was generated with the Pavian R package version 0.8.4 on Fri May 28 20:31:10 2021. Please cite Pavian if you use it in your research.
