# Supplementary material for: Single-cell genomics for resolution of conserved bacterial genes and mobile genetic elements of the human intestinal microbiota using flow cytometry
Source: Gut Microbes. 2022 Feb 7;14(1):2029673. doi: 10.1080/19490976.2022.2029673 (PMC8824198; doi:10.1080/19490976.2022.2029673)
Supplement: Supplemental Material [file KGMI_A_2029673_SM1460.zip › supplementary/Faecalibacterium_MMSeqs_report.html]

Uploaded\_sample\_set-report.utf8.md


# Classification report for Uploaded sample set

#### Pavian R package v0.8.4

#### Fri May 28 20:34:12 2021

# Sample set summary

- Classification summary
- Raw read numbers
- Sample information

# Classification results

- Bacteria
- Viruses
- Eukaryotes
- Eukaryotes/Fungi
- Eukaryotes/Protists

Showing 100 of 6354 species.

# Sankey visualization

## 1p2\_10A

## 1p2\_10G

## 1p2\_12C

## 1p2\_4B

## 1p2\_5E

## 1p5\_10C

## 1p5\_11F

## 1p5\_12F

## 1p5\_3B

## 1p5\_4H

## 1p5\_8H

## 2p1\_11H

## 2p1\_9A

## 2p1\_9C

## 2p1\_9H

## 3p2\_2D

## 3p2\_2G

## 3p2\_4G

## 3p2\_6A

## 3p2\_9B

## 5p5\_10B

## 5p5\_12D

## 5p5\_5B

## 5p5\_5D

## 8p5\_11B

## 8p5\_5B

## 8p5\_8D

# About

This file was generated with the Pavian R package version 0.8.4 on Fri May 28 20:34:16 2021. Please cite Pavian if you use it in your research.
