# Supplementary material for: Single-cell genomics for resolution of conserved bacterial genes and mobile genetic elements of the human intestinal microbiota using flow cytometry
Source: Gut Microbes. 2022 Feb 7;14(1):2029673. doi: 10.1080/19490976.2022.2029673 (PMC8824198; doi:10.1080/19490976.2022.2029673)
Supplement: Supplemental Material [file KGMI_A_2029673_SM1460.zip › supplementary/Mix_MMSeqs_Report.html]

Uploaded\_sample\_set-report.utf8.md


# Classification report for Uploaded sample set

#### Pavian R package v0.8.4

#### Fri May 28 20:47:02 2021

# Sample set summary

- Classification summary
- Raw read numbers
- Sample information

# Classification results

- Bacteria
- Viruses
- Eukaryotes
- Eukaryotes/Fungi
- Eukaryotes/Protists

Showing 100 of 9588 species.

# Sankey visualization

## 1p2\_4H

## 1p2\_8B

## 1p2\_8F

## 1p5\_6B

## 2p1\_6B

## 3p2\_11F

## 3p2\_4H

## 3p2\_9F

## 5p4\_10E

## 5p4\_11E

## 5p4\_11H

## 5p4\_12F

## 5p4\_4A

## 5p4\_7G

## 6p4\_10D

## 6p4\_12C

## 6p4\_2A

## 6p4\_2D

## 6p4\_4F

## 6p4\_5G

## 6p4\_7D

## 6p4\_7G

## 6p4\_8A

## 6p4\_8H

## 6p5\_11D

## 6p5\_2E

## 6p5\_3A

## 6p5\_3C

## 6p5\_3D

## 6p5\_4F

## 6p5\_8F

## 6p5\_8G

## 7p5\_2E

## 7p5\_3B

## 7p5\_3C

## 7p5\_6A

## 7p5\_6G

## 7p5\_9F

# About

This file was generated with the Pavian R package version 0.8.4 on Fri May 28 20:47:07 2021. Please cite Pavian if you use it in your research.
